# Survey on the research direction of EEG-based signal processing

**DOI:** 10.3389/fnins.2023.1203059

**Published:** 2023-07-13

**Authors:** Congzhong Sun, Chaozhou Mou

**Affiliations:** School of Mathematics and Statistics, Shandong University, Weihai, China

**Keywords:** electroencephalography (EEG), brain-computer interface (BCI), preprocessing, feature extraction, classification, deep learning (DL), multi-method fusion

## Abstract

Electroencephalography (EEG) is increasingly important in Brain-Computer Interface (BCI) systems due to its portability and simplicity. In this paper, we provide a comprehensive review of research on EEG signal processing techniques since 2021, with a focus on preprocessing, feature extraction, and classification methods. We analyzed 61 research articles retrieved from academic search engines, including CNKI, PubMed, Nature, IEEE Xplore, and Science Direct. For preprocessing, we focus on innovatively proposed preprocessing methods, channel selection, and data augmentation. Data augmentation is classified into conventional methods (sliding windows, segmentation and recombination, and noise injection) and deep learning methods [Generative Adversarial Networks (GAN) and Variation AutoEncoder (VAE)]. We also pay attention to the application of deep learning, and multi-method fusion approaches, including both conventional algorithm fusion and fusion between conventional algorithms and deep learning. Our analysis identifies 35 (57.4%), 18 (29.5%), and 37 (60.7%) studies in the directions of preprocessing, feature extraction, and classification, respectively. We find that preprocessing methods have become widely used in EEG classification (96.7% of reviewed papers) and comparative experiments have been conducted in some studies to validate preprocessing. We also discussed the adoption of channel selection and data augmentation and concluded several mentionable matters about data augmentation. Furthermore, deep learning methods have shown great promise in EEG classification, with Convolutional Neural Networks (CNNs) being the main structure of deep neural networks (92.3% of deep learning papers). We summarize and analyze several innovative neural networks, including CNNs and multi-structure fusion. However, we also identified several problems and limitations of current deep learning techniques in EEG classification, including inappropriate input, low cross-subject accuracy, unbalanced between parameters and time costs, and a lack of interpretability. Finally, we highlight the emerging trend of multi-method fusion approaches (49.2% of reviewed papers) and analyze the data and some examples. We also provide insights into some challenges of multi-method fusion. Our review lays a foundation for future studies to improve EEG classification performance.

## 1. Introduction

The Brain-Computer Interface (BCI) is a communication system that allows humans to send messages and commands to the outside world without relying on peripheral nerves and muscles (Wolpaw et al., 2000). The BCI system is composed of four primary components: signal acquisition, signal processing, control equipment, and feedback link. Signal acquisition technology in BCI systems can be divided into two categories: non-invasive methods and invasive methods. While invasive methods, such as implanting electrodes directly into the brain, have been explored in some studies, they are not commonly used due to the potential risks and complicated operations. Thus, non-invasive methods are used in many studies. Non-invasive methods include functional Magnetic Resonance Imaging (fMRI), functional Near-Infrared Spectroscopy (fNIRS), Magnetoencephalography (MEG), Electroencephalography (EEG), and Positron Emission Tomography (PET).

Among the aforementioned methods, EEG signals are commonly utilized due to their features of safety, portability, ease of use, high temporal resolution, and low cost (Singh et al., 2021). EEG signals are a useful tool for directly reflecting the activities of the brain, in both BCI and clinical applications (Wolpaw et al., 2000; Michel and Murray, 2012). For example, a typical application of EEG-BCI systems is to control a robot arm by brain signals, which will be greatly helpful for not only the disabled but also general people to improve their life (Jeong et al., 2020). Therefore, it is important to process and analyze EEG signals so as to fit multitudes of applications in BCI. EEG signal processing typically involves three main steps: preprocessing, feature extraction, and classification.

EEG signals are typically collected using multiple electrodes placed on the scalp, with electrodes placed on different scalp locations to collect signals from various brain areas. The positioning or arrangement of electrodes is called the montage, and there are two broad categories of montages: bipolar and referential (Kumar and Bhuvaneswari, 2012). The former compares an electrode with its neighbors and outputs their difference as a channel, while the latter chooses one reference electrode and compares all other electrodes with this electrode (Sanei and Chambers, 2021). After acquisition, the raw EEG signals are represented as 2D tensors (multi-channel 1D sequences) with shape *C* × *T*, where *C* and *T* represent the number of channels and time samples, respectively. Many datasets adopt referential montage, and thus in those datasets, one channel corresponds to one electrode. The collected signal can be considered as complex mixtures of the activities of many brain cells, resulting in EEG signals exhibiting various rhythms that reflect diverse cognitive states and are associated with different brain activities. Different rhythms can be broadly categorized into several bands based on frequency, including delta (1–4 Hz), theta (4–8 Hz), alpha (8–12 Hz), beta (13–25 Hz), and gamma (≥25 Hz; Kumar and Bhuvaneswari, 2012; Singh et al., 2021). Additionally, EEG signals can have different paradigms, referring to different types of tasks or stimuli. Common paradigms include P300 (Bashashati et al., 2007), Motor Imagery (MI; Cano-Izquierdo et al., 2012), Steady-State Visual Evoked Potential (SSVEP; Wolpaw et al., 2003), etc. These paradigms often relate to specific brain activities and signal processing tasks. For example, when a person imagines his/her limb moving, some specific changes in EEG signals will occur, the paradigm of which is called MI, and this will relate to the task of controlling a device to move.

However, in order to effectively process EEG signals, it is important to consider some of their inherent characteristics, including:

1. Low spatial resolution and low Signal-to-Noise Ratio (SNR). EEG signals are susceptible to interference and artifacts. Therefore, signal processing must address the challenges of separating noise from abnormal signals and extracting meaningful features.
2. Dimensionality disaster. EEG signals have multiple channels during acquisition, leading to exponentially increasing computation as dimensionality increases.
3. Non-stationariness. The statistics of EEG signals change rapidly over time.
4. Lack of large labeled training samples. Due to the requirement for high participant focus during data acquisition, it is difficult to obtain a large number of brain data. For example, frequent visual stimulation during Visual Evoked Potential (VEP) signals acquisition can cause visual fatigue. Consequently, many datasets have a limited number of samples.
5. Subject-specificity. EEG signals differ significantly among individuals, leading to poor stability and generalization. Models trained on specific subjects may not perform well on new subjects (Lashgari et al., 2020).

Furthermore, unlike image processing and natural language processing, we lack specific knowledge of the physiological activity of our brains. This means that we cannot intuitively understand EEG signals or apply our a priori knowledge to them.

The rest of this article is organized as follows: In the next part of Section 1, we introduce three steps of EEG signal processing and summarize several previous reviews. In Section 2, we summarize the relevant information for the proposed approaches and how the papers were selected and assessed. In Section 3, discussions are introduced, where specific methods are compared, including preprocessing, deep learning, and multi-method fusion. In Section 4 we show our conclusions finally.

### 1.1. Preprocessing

After collecting EEG signals, it is necessary to preprocess the data in order to remove irrelevant noise and reduce computational complexity. In the following text, we will introduce some preprocessing methods.

#### 1.1.1. Basic preprocessing methods

Basic preprocessing methods are based on some basic characteristics of EEG signals. These methods including filtering, electrode positioning, deletion of useless data, baseline correction, heavy reference, downsampling, removal of artifacts, removal of bad segments, etc. These methods can be easily invoked by the EEGLAB toolbox, a useful Matlab toolbox that facilitates various preprocessing operations of EEG signals (Delorme and Makeig, 2004; Bashashati et al., 2007).

Filtering is one of the most frequently used preprocessing methods. EEG signals have low SNR and different rhythms; thus, band-pass filtering is suitable to eliminate noise that has a different frequency from EEG signals and separate useful rhythms from the source (Saeidi et al., 2021).

#### 1.1.2. Data augmentation

To address the problem of small dataset, data augmentation is an effective method, which includes both non-deep learning methods like sliding windows, noise injection, and segmentation and recombination, as well as deep learning methods like Generative Adversarial Networks (GAN) and Variation AutoEncoder (VAE; Lashgari et al., 2020; He et al., 2021). Many models, especially deep learning models, require a large amount of training data to achieve high classification accuracy and avoid overfitting. However, collecting a large number of EEG data is difficult due to the intrinsic characteristics of EEG signals. Data augmentation can generate new data from a small dataset, providing enough training data.

Among non-deep learning methods, sliding window crops the signals into several segments by sliding a window on the signals. The length and overlap of segments depend on the window size and window step. Sliding window increases the number of training data but also eliminates long-term information. Segmentation methods can cut out specific time intervals based on the temporal characteristics of EEG signals (Lu et al., 2022). Gaussian noise injection injects a random matrix from a Gaussian distribution into the original data to achieve data augmentation (Okafor et al., 2017). These methods are intuitive and simple, but they may exacerbate the overfitting of models due to their similarity after augmentation.

GAN and its variants can generate artificial data by training a generative network and a discriminative network (Zhang A. et al., 2021). The generative network accepts random noise from a specific distribution (e.g., Gaussian) and attempts to generate synthetic data similar to real data, while the discriminative network is trained to classify the real and synthetic data. These two networks are adversarial, and after adequate training, the generative network will produce verisimilar signals. For VAE, like a normal autoencoder (which will be introduced below), the encoder converts the raw data into latent data, and the decoder maps the latent data back to real data. To generate new data, the VAE randomly samples points from the learned latent space, and then passes these samples through the decoder network, which reconstructs them into new samples. Both GAN and VAE generate new samples indirectly.

#### 1.1.3. Channel selection

During acquisition, every electrode records a channel of data. Thus the raw EEG signals has *C* channels, which is known as the multi-dimensionality of EEG signals. Different channels correspond to different areas of the brain. For a specific task, some channels may contain task-irrelevant or redundant information (Liu et al., 2016), which increases data size and time cost, and can negatively impact the performance of BCI (Asensio-Cubero et al., 2013). Channel selection is a method to select the most salient channels of task-related regions as the optimal channels so as to improving performance and efficiency. However, multichannel EEG data contain complex channel correlations rather than simple adjacencies (Cona et al., 2009; Hamedi et al., 2016). Therefore, we should seek selection criteria based on the features of channels, such as correlation, electrode distance, and task characteristics, to select the channels that preserve the signal features to the maximum extent possible.

#### 1.1.4. Dimensionality reduction

EEG signals are multi-dimensional signals. Compared to traditional 1D signals, EEG signal processing has high computational complexity. Therefore, we usually need to impose corresponding constraint assumptions according to the structure of EEG signals and reduce the dimensionality to further improve the extraction effect and classification robustness of feature signals.

Many algorithms can reduce the dimension. For instance, Principle Component Analysis (PCA) can decompose the EEG signal into linearly uncorrelated components which have the maximum variance. Redundant components such as interference from eyes and muscles can be separated by PCA before reconstructing the EEG signal (Liu and Yao, 2006). Independent Component Analysis (ICA) separates artifacts from EEG signals as independent components based on data features (Saeidi et al., 2021). Geng et al. (2022) proposed that preprocessing can decompose complex mixed signals into independent signals by ICA to achieve separation of P300 signals from noise. However, because the ICA algorithm was not trained to learn the characteristics of noise signals, some valuable signals may be removed as noise, causing some brain activity information loss. By using the Wavelet Transform (WT), the feature of EEG signals can be extracted, and then by ICA-WT filtering, the noise artifacts can be effectively eliminated, thus effectively improving the accuracy of EEG signals of different subjects (Ayoobi and Sadeghian, 2022). Ayoobi and Sadeghian (2022) also investigated the AutoEncoder (AE) for preprocessing, where the encoder extracts the information of the input raw data into a small latent space and then decodes the latent data to reconstruct the dataset. Since the latent variables carry information of raw signals but have fewer dimensions, we can use latent variables as the input of subsequent steps.

### 1.2. Feature extraction

Feature extraction generally refers to the extraction of hidden brain information features from signals. Next, we will introduce some feature extraction methods, including both conventional algorithms (non-deep learning) and deep learning algorithms.

#### 1.2.1. Feature extraction by conventional algorithms

The conventional algorithms adopted in feature extraction include Common Spatial Pattern (CSP), Fourier Transform (FT), Power Spectral Density (PSD), Wavelet Transform (WT), Wavelet Packet Decomposition (WPD), Empirical Mode Decomposition (EMD), Autoregression (AR), and Hjorth parameters, etc. (Wolpaw et al., 2003; Kim et al., 2018; Torres et al., 2020).

Common Spatial Pattern (CSP) is a space domain filtering algorithm used for binary classification tasks. CSP extracts the spatial distribution components of each class of the multi-channel EEG signal and seeks the best projection direction to maximize the variance of one class and minimize that of the other class (Meng et al., 2022). Since CSP maximizes the difference among EEG signals, it is more capable of mining the features of EEG signals. However, the number of electrodes needs to be further optimized because a large number of electrodes are required for multichannel analysis of EEG signals. There are also many variants of CSP, such as Common Space Spectral Pattern (CSSP), Filter Bank CSP (FBCSP), etc. (Park et al., 2018; Maruyama et al., 2020; Kumar et al., 2021).

EEG signals exhibit various frequency bands, each associated with distinct brain activities. Therefore, analyzing EEG signals in the frequency domain and time-frequency domain is a common approach. Fourier Transform (FT), particularly Fast Fourier Transform (FFT) as a fast version, is a fundamental tool in frequency analysis. It can transform stationary signals to the frequency domain to extract frequency features. Power Spectral Density (PSD), the FT of the autocorrelation function of a signal, reveals the power (energy) distribution across different frequencies. However, FT and PSD can only analyze the frequency content across the entire series. To analyze the frequency changes over time, time-frequency analysis methods are necessary. Short-Time Fourier Transform (STFT) segments signals into short time intervals before applying Fourier transform to analyze the frequency variance. Wavelet Transform (WT), an improved method of STFT, is suitable for analyzing non-stationary signals such as EEG signals (Al-Fahoum and Al-Fraihat, 2014). Wavelet Packet Decomposition (WPD), a modification of WT, further decomposes the high-frequency sub-bands and provides better frequency resolution. Following the time-frequency analysis, the signal shape becomes *F* × *T* × *C*, where *F* represents the frequency resolution.

Furthermore, Autoregression (AR) is a popular approach used in time-series prediction. AR assumes that the current value of a time series depends linearly on its past values (Saeidi et al., 2021). Another technique used to analyze non-stationary EEG signals is Empirical Mode Decomposition (EMD). EMD is a non-linear method that decomposes a signal into its intrinsic modes of oscillation (El-Kafrawy et al., 2014). This method has been used to study the time-frequency characteristics of EEG signals. Finally, Hjorth parameters are statistical features used to extract information about EEG signals (Du et al., 2021). The three Hjorth parameters are Activity, Mobility, and Complexity. Activity measures the signal's energy, Mobility measures its frequency content, and Complexity reflects the signal's nonlinearity.

#### 1.2.2. Automatic feature extraction by deep learning

Traditional feature extraction algorithms such as CSP and PSD have limitations. For instance, feature extraction and classification are performed separately, and much experience or priori knowledge is manually added during feature extraction. In contrast, deep learning algorithms utilize a deep architecture consisting of many hidden layers to automatically extract spatiotemporal features of EEG signals using a large number of training parameters and data (Aellen et al., 2021). The location of discriminative patterns of deep learning in spatial detection is irrelevant, which often leads to neural networks outperforming conventional machine learning algorithms. Deep learning algorithms are capable of learning useful features that capture the underlying structure of EEG signals, without the need for explicit feature extraction. Furthermore, deep learning methods have the potential to overcome the limitations of traditional feature extraction methods and to enable more accurate classification of EEG signals.

### 1.3. Classification algorithms

General tasks of machine learning can be sorted into two categories: regression and classification. There are several novel papers adopting regression methods, such as Jeong et al. (2020) which adopted deep learning to track the movement of a robot arm. But among the research on EEG, most studies focused on classification, since the labels of tasks and outputs of processing are usually categorical variables. The development of higher performance and more robust classification algorithms is a key focus in EEG research. The selection of a classification algorithm plays a crucial role in determining the performance of the system.

#### 1.3.1. Conventional classification algorithms

Conventional BCI classification algorithms include Support Vector Machine (SVM; Li et al., 2018), Linear Discriminant Analysis (LDA; Vidaurre et al., 2011), and *k*-Nearest Neighbor (KNN; Tang et al., 2019). SVM can be used for linearly separable data by finding an optimal hyperplane through optimization algorithms. For linearly inseparable problems, a kernel function can be used to transform the data into a higher dimensional space. LDA is a simple linear classifier that projects all samples onto a line to maximize the inter-class distance and minimize the intra-class variance. KNN is a method for classification that counts the class number of the *k* nearest samples with the least distance to the new sample.

While SVM and LDA are popular algorithms with good performance, SVM can be computationally complex and LDA requires linear separability. KNN is simple and easy to use, but can be weak in generalization.

#### 1.3.2. Deep learning algorithm

**Figure 1:**
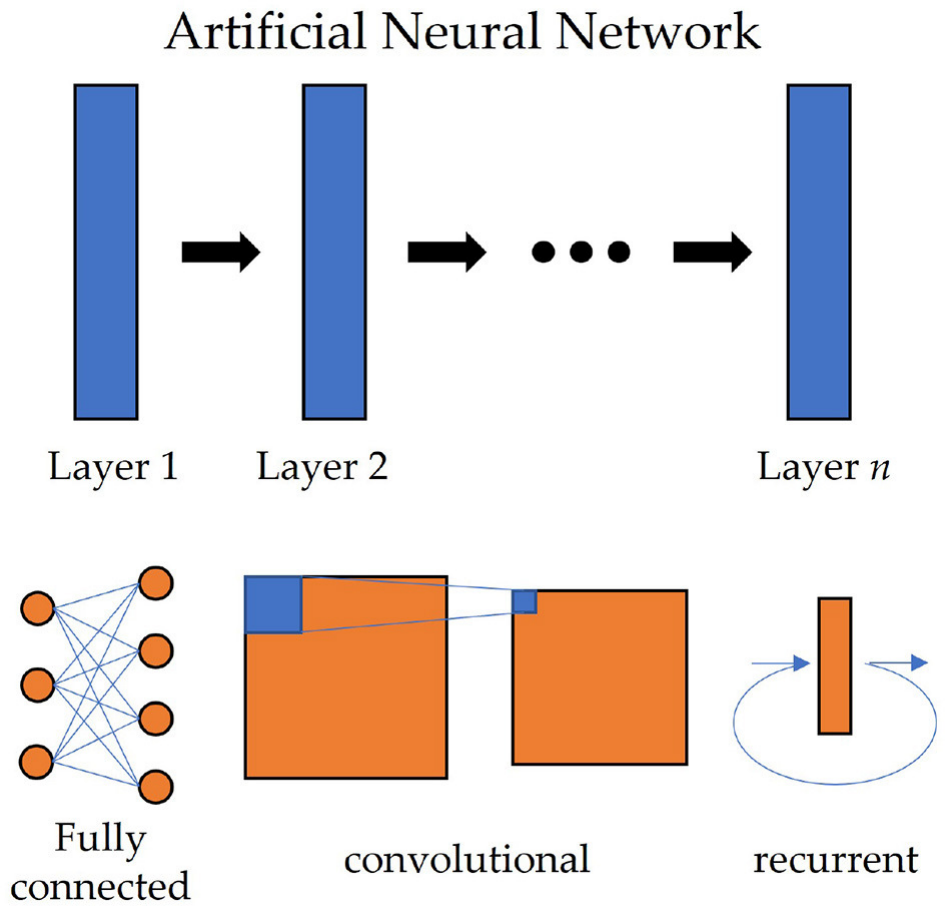
The structure of artificial neural networks in deep learning. Each layer in the network can be an FC layer **(left bottom)**, a convolutional layer of CNN **(middle bottom)**, or a recurrent layer of RNN **(right bottom)**.

Deep learning algorithms have been shown to be effective in extracting features from high-dimensional data. They are particularly useful for processing EEG signals, which are often high-dimensional and complex. Deep learning methods use Artificial Neural Networks (ANN) to process data, which can automatically learn features that are relevant to the task, and can generalize well across different tasks. The structure of ANN is shown at the top of Figure 1. Common deep learning algorithms and ANNs applied to EEG signal processing include Multilayer Perceptrons (MLP), Convolutional Neural Network (CNN; Mane et al., 2020), Recurrent Neural Network (RNN; Luo et al., 2018), etc.

The most simple deep learning algorithm is the Multilayer Perceptron (MLP). An MLP is a network constructed by Fully Connected (FC) layers (also called Dense layer or Linear layer in some papers), in which a linear transform and a subsequent nonlinear activation function are used sequentially. Let ***x*** ∈ ℝ^*m*^ be the input, the output ***y*** ∈ ℝ^*n*^ of a single FC layer is calculated by


(1) $$\begin{array}{l} y=\sigma \left(Wx+b\right); \end{array}$$

where ***W*** ∈ ℝ^*m* × *n*^, ***b*** ∈ ℝ^*n*^ and σ are weight matrix, bias and activation function, respectively (Zhang A. et al., 2021). The bottom left of Figure 1 illustrates an example of the FC layer. MLP is seldom used alone now, but FC is often combined with other networks, used as the last layer to classify the features.

One deep learning algorithm that has been widely used in EEG signal processing is the Convolutional Neural Network (CNN). CNN adopts convolution operations to automatically extract features from the data. CNN consists of multiple convolutional layers, which accept a 2D image or a 3D multi-channel image as the input and apply convolution operation on it. For a given input ***X*** ∈ ℝ^*c* × *m* × *n*^, a convolutional layer gives the output ***Y*** ∈ ℝ^*d* × *m*^′ × *n*′ by


(2) $$\begin{array}{l} Y_{d,i,j}=\overset{\delta_{1}}{\underset{p=-\delta_{1}}{\sum }}\overset{\delta_{2}}{\underset{q=-\delta_{2}}{\sum }}\overset{c}{\underset{r=1}{\sum }}K_{d,r,p,q}X_{r,i+p,j+q}; \end{array}$$

where *c* and *d* denotes the input and output channels, respectively; $K\in {\mathbb{R}}^{d\times c\times \left(2\delta_{1}+1\right)\times \left(2\delta_{2}+1\right)}$ is the kernel and also parameters of CNN (Zhang A. et al., 2021). A graph of convolutional layers is shown at the bottom middle of Figure 1. Since *c* and *d* are decided by the layers, the shape of kernel ***K*** is often shorted to (2δ_1_+1) × (2δ_2_+1). The operation of a convolution layer is actually a spatial filter, but its parameters can be updated by the backpropagation algorithm automatically. Also, different sizes of kernels can be used for different tasks. When the input tensor has size *C* × *T*, like raw EEG signals, if we set the size of the kernel to 1 × *n*, it will extract the features along the time dimension; if *m* × 1, it will seek the correlations of different channels.

Plenty of CNN structures have been applied to EEG signal processing, such as residual network (ResNet; He et al., 2016) and ConvNet (Azizpour et al., 2016). EEG-specific neural networks have also been proposed, such as EEGNet. EEGNet is a compact CNN with only three convolutional layers, and it adopts two special structures—depthwise convolutional layers and separable convolutional layers to reduce the number of parameters and computational costs (Lawhern et al., 2018). EEGNet is proposed in 2018, and has been shown to be more robust, more compact, and less data-intensive to different paradigms of EEG signals, and thus has widespread application in EEG signal processing.

Recurrent Neural Networks (RNN) are another type of deep learning algorithm that has been used in EEG signal processing. RNN appends a hidden state into conventional MLP and passes the hidden state into the next unit, which is fit for extracting long-term relations of time series models. EEG signals, as a kind of context-sensitive sequences, are also suitable for using RNN to extract temporal features. A recurrent unit of RNN accepts a sequence ***x*** ∈ ℝ^*T*^ as input, and will calculate the hidden state ***h*** and output ***y*** by


(3) $$\begin{array}{l} \{\begin{array}{l} h_{t}=\sigma (W_{1}x_{t}+W_{2}h_{t-1}+b_{2});\\ y_{t}=W_{3}h_{t}+b_{2}; \end{array} \end{array}$$

where ***W***_1_, ***W***_2_, ***W***_3_ are weight matrices, ***b***_1_, ***b***_2_ are biases and σ is the activation function. The structure of a recurrent layer is illustrated in the bottom right of Figure 1. Long Short-Term Memory (LSTM) network (Wang et al., 2018) and Gated Recurrent Unit (GRU; Nakagome et al., 2020) are two popular variants of RNN. By appending several gate units into conventional RNN, they inherit the advantages of RNN and lead to more accurate analysis of sequence data.

Batch Normalization (BN), dropout, and attention mechanisms are also spreadly used. During the mini-batch feedforward step, the data passing BN will subtract the mean and divided by the variance, converted into zero mean and unit variance (Zhang A. et al., 2021). BN is usually used before an activation function so as to improve the distinction of the activation function. Dropout is a method that makes the unit of networks stop with a probability *p* to avoid overfitting, which is only used during training (Zhang A. et al., 2021). Attention mechanisms simulate the attention functions of our brain to make significant information prominent. Attention mechanisms are not a single algorithm, but a set of methods including conventional methods and CNN-based methods.

In addition, the Transfer Learning (TL) algorithm supplements the limited training data with data transferred from other domains to improve the system portability and solve the problem of long training time while ensuring accuracy (Zhang et al., 2020). TL uses the similarities between two tasks, and transfers what has been learned from the source network into the target network to enhance the model. Facing a small dataset, TL has become an effective method to improve performance.

### 1.4. Research issues and contribution

In previous research, Alzahab et al. (2021) provided a comprehensive summary of hybrid deep learning algorithms used in EEG-based BCI systems between 2015 and 2020 and compared their accuracy. However, they also highlighted the lack of evidence regarding the impact of preprocessing on the accuracy of EEG classification. Vallabhaneni et al. (2021) surveyed articles that used deep learning to decode EEG signals in different applications, and outlined some existing deep learning problems. Chen and Xie (2019) suggested that the choice of data processing methods should be based on the characteristics and size of the EEG signals. He et al. (2021) reviewed the application of data augmentation in EEG and found that it could improve classification performance and overcome the challenges of small-scale datasets. Saeidi et al. (2021) systematically reviewed the machine learning-based EEG decoding methods in terms of different tasks and concluded that CNN, SVM, and WT become the most effective deep learning, conventional machine learning, and feature extraction methods. These studies have contributed to the advancement of BCI-EEG and highlighted the existing challenges in this field.

In this paper, we analyze the latest studies since 2021 and compare them to the reviews and articles before 2020. We choose post-2021 articles since there have been plenty of reviews focused on the articles before 2020, and we want to summarize the latest development. Based on the articles we reviewed, we provide an overview of the research landscape in EEG-based BCI signals and the emerging trends. We focus on answering the following research questions: What are the current research topics in BCI signal processing? How can we evaluate BCI performance and improve it? What are the innovative approaches being explored in this field? We also evaluate the strengths and limitations of different methods. Our main contributions are as follows:

1. We summarize various methods of EEG-based signal processing of BCI systems and the major research trends. We also propose solutions to potential issues.
2. We attempt to seek valid, reasonable, and useful indicators of BCI performance.
3. We confirm the effectiveness of preprocessing on the performance.
4. We discuss deep learning and multi-method fusion studies in different aspects and summarize several existing problems.

## 2. Search methods and reviewed table

### 2.1. Search methods

**Figure 2:**
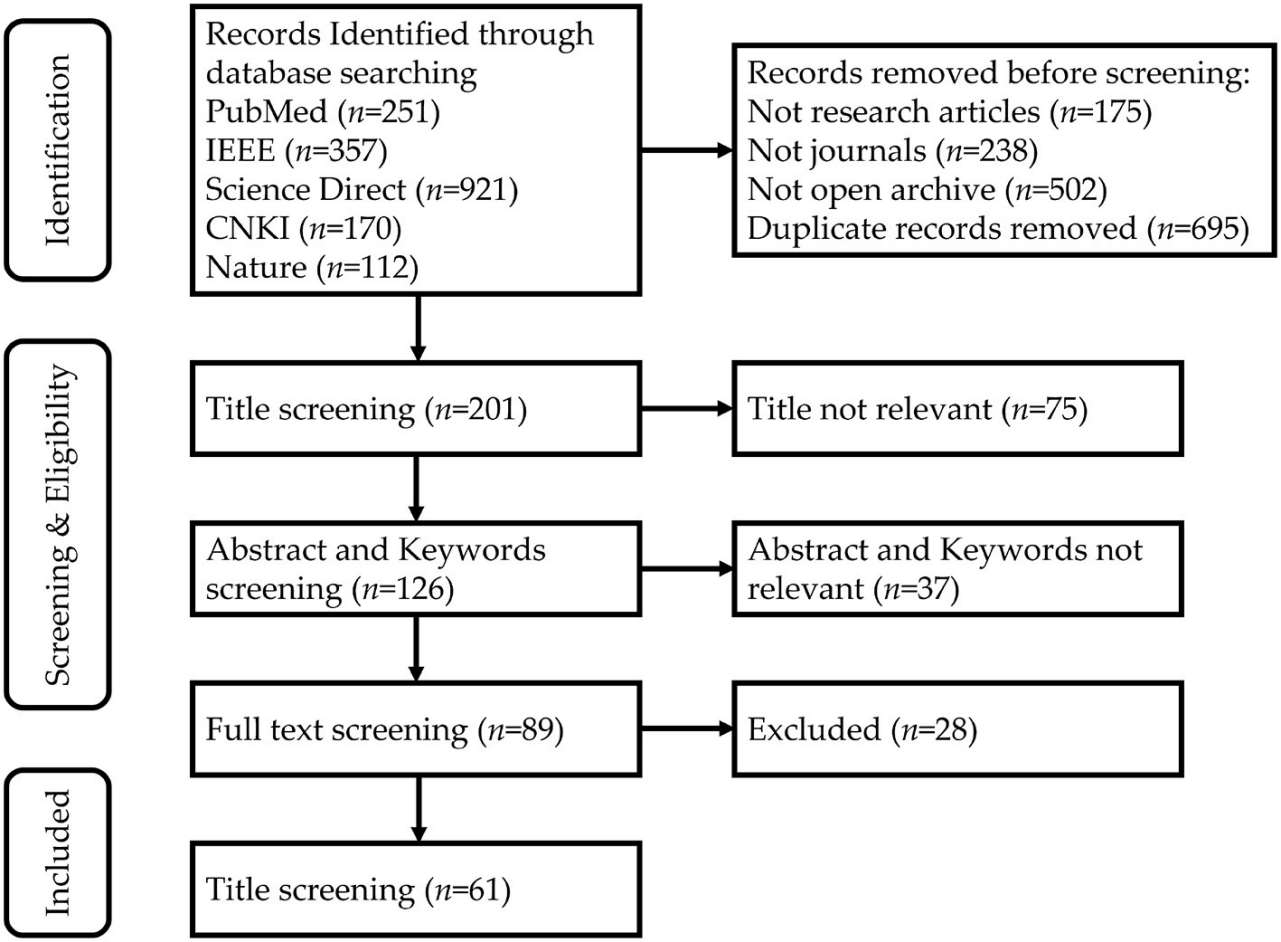
The search method for identifying studies about EEG signal processing.

Research articles were selected for review on 31 June 2022. The following databases were conducted: CNKI, PubMed, Nature, IEEE Xplore, and Science Direct. The search covered studies published between 2021 and 2022. The following query terms are used: (“brain-machine interface” OR “brain-computer interface” OR “BCI”) AND (“EEG” OR “electroencephalography”) AND (“preprocessing” OR “feature extraction” OR “classification”). This search resulted in 61 research papers, as shown in Figure 2.

### 2.2. Reviewed papers

By collecting and summarizing the papers on EEG-based BCI signal processing, we sort out a variety of new methods for EEG signal processing and analyze the characteristics of their performance.

Since the table of all the reviewed papers is too large, we only provide a list of the papers reviewed in this article. A detailed table with information on the directions and performance of each paper can be found in the Supplementary material. The following reviewed papers are presented in ascending order of their published date (Aellen et al., 2021; Asheri et al., 2021; Ashwini and Nagaraj, 2021; Awais et al., 2021; Cai et al., 2021; Dagdevir and Tokmakci, 2021; De Venuto and Mezzina, 2021; Du et al., 2021; Fan et al., 2021, 2022; Ferracuti et al., 2021; Gao N. et al., 2021; Gao Z. et al., 2021; Gaur et al., 2021; Lashgari et al., 2021; Lian et al., 2021; Liu and Jin, 2021; Liu and Yang, 2021; Liu et al., 2021; Qi et al., 2021; Rashid et al., 2021; Sun et al., 2021; Varsehi and Firoozabadi, 2021; Vega et al., 2021; Vorontsova et al., 2021; Wahid and Tafreshi, 2021; Wang and Quan, 2021; Xu C. et al., 2021; Xu F. et al., 2021; Yin et al., 2021; Zhang K. et al., 2021; Zhang Y. et al., 2021; Algarni et al., 2022; Ali et al., 2022; Asadzadeh et al., 2022; Ayoobi and Sadeghian, 2022; Bagchi and Bathula, 2022; Chang et al., 2022; Chen J. et al., 2022; Chen L. et al., 2022; Cui et al., 2022; Geng et al., 2022; Islam et al., 2022; Jia et al., 2022; Kim et al., 2022; Ko et al., 2022; Li and Sun, 2022; Li H. et al., 2022; Lin et al., 2022; Li Q. et al., 2022; Lu et al., 2022; Ma et al., 2022; Mattioli et al., 2022; Meng et al., 2022; Pei et al., 2022; Song et al., 2022; Suhaimi et al., 2022; Tang et al., 2022; Xu et al., 2022; Ying et al., 2022; Zhao et al., 2022).

## 3. Results and discussion

The BCI system based on EEG signals analyzes the instructions issued by the human brain. The processing and classification of EEG signals determine the performance of the BCI system. In the study of EEG signal processing, several questions arise, including:

1. What is the current development trend of EEG signal processing techniques?
2. How to select a processing method suitable for EEG signal characteristics?
3. How to apply deep learning algorithm properly to enhance performance?
4. Why do we need multi-method fusion, and are they valid?

Considering the characteristics of EEG signals, the processing of EEG signals aims to find a feasible method to fuse the signal pattern to seek high-performance and strong applicability processing methods. Currently, many preprocessing, feature extraction, and classification algorithms are applied to EEG signals, each with its own advantages and disadvantages. Therefore, appropriate methods should be selected according to specific situations. In this paper, we classify algorithms according to the categories introduced in Section 1. In the following, we will discuss the directions and numbers of reviewed studies.

**Figure 3:**
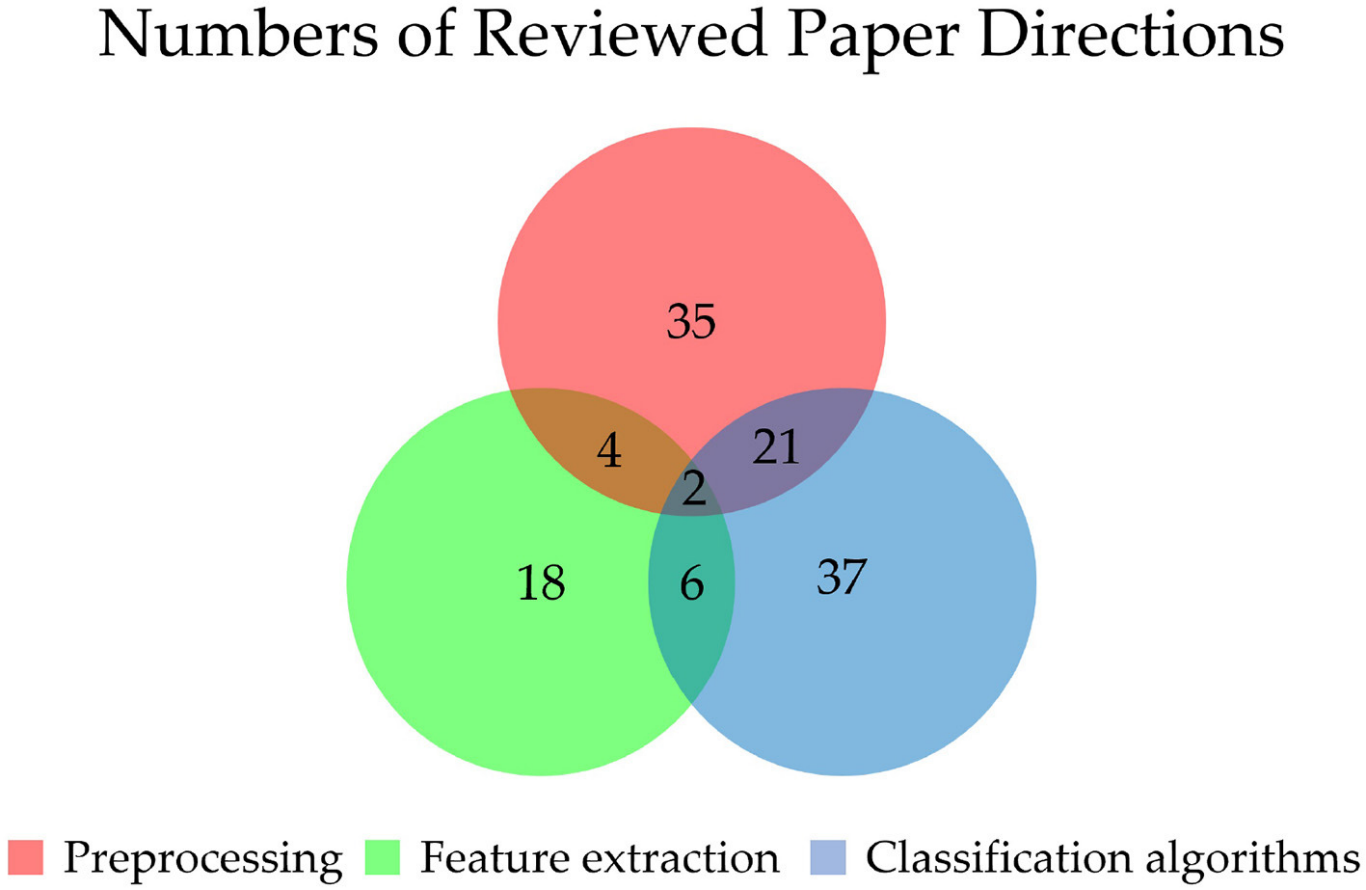
Numbers of different directions of EEG signal processing. The numbers of preprocessing (P), feature extraction (F), and classification algorithm (C) papers are 35, 18, and 37, respectively. The numbers of papers about both P and F, both P and C, and both F and C (all including two papers in the center) are 4, 21, and 6, respectively. There are two papers focus on all three directions.

For preprocessing, by increasing data samples and identifying valid data, it can obtain better features of signals and reduce computation costs. By studying the 61papers, we found that the number of preprocessing studies is 35 (57.4%). This suggests that preprocessing is an important step to seek more prominent features and achieve higher performance of EEG signals. As for feature extraction, since deep learning has a strong ability in automatic feature extraction learning of EEG signals, feature extraction tends to be completed automatically by deep learning algorithms. Therefore the number of feature extraction studies is only 18 (29.5%). The number of classification algorithms is 37 (60.7%), indicating that the innovation of classification algorithms is still the main method to improve the performance of BCI. Thus it is the focus and hotspot of current EEG processing research. The study number of these three directions is shown in Figure 3.

### 3.1. Indicators of EEG

Various indicators are used to measure the performance of classifiers in EEG classification. Accuracy (ACC) remains the most widely used indicator across papers as it provides an intuitive measure of classification performance. However, several other indicators, such as the confusion matrix (Algarni et al., 2022) and kappa (Lian et al., 2021), are also commonly employed. The confusion matrix compares the number of predicted and actual labels in an *n* × *n* matrix. On the other hand, kappa measures the consistency of classification and is used in multiple studies to gauge performance improvement.

Although accuracy is the primary indicator, 16 papers also compare other factors like parameter number, Information Translating Rate (ITR), and computation time. Parameter number is used to measure the complexity of a model, especially for neural networks. ITR and computation time reflect the speed of classification and are crucial factors in many studies. Notably, six studies improve ITR, with the maximum ITR reaching 170.67 bit/min in Zhang K. et al. (2021). Seven studies achieve time cost reduction, reflecting the importance of computational efficiency.

Given the subject-specificity of EEG signals, researchers also investigate the variation between subject-dependent (within-subject) and subject-independent (cross-subject) accuracy. Subject-independent tasks involve testing models on new individuals whose data are not included in the training data, whereas subject-dependent tasks test on different segments of the same individuals' data. Singh et al. (2021) has highlighted the importance of subject-independent tasks— they play a crucial role in designing a plug-and-play calibration-free BCI device. While many studies report high within-subject accuracy, which can exceed 90% in several cases, cross-subject accuracy is still lower (mostly around 50%; Lashgari et al., 2021; Fan et al., 2022). Improving cross-subject accuracy has become a significant direction.

### 3.2. Preprocessing

**Figure 4:**
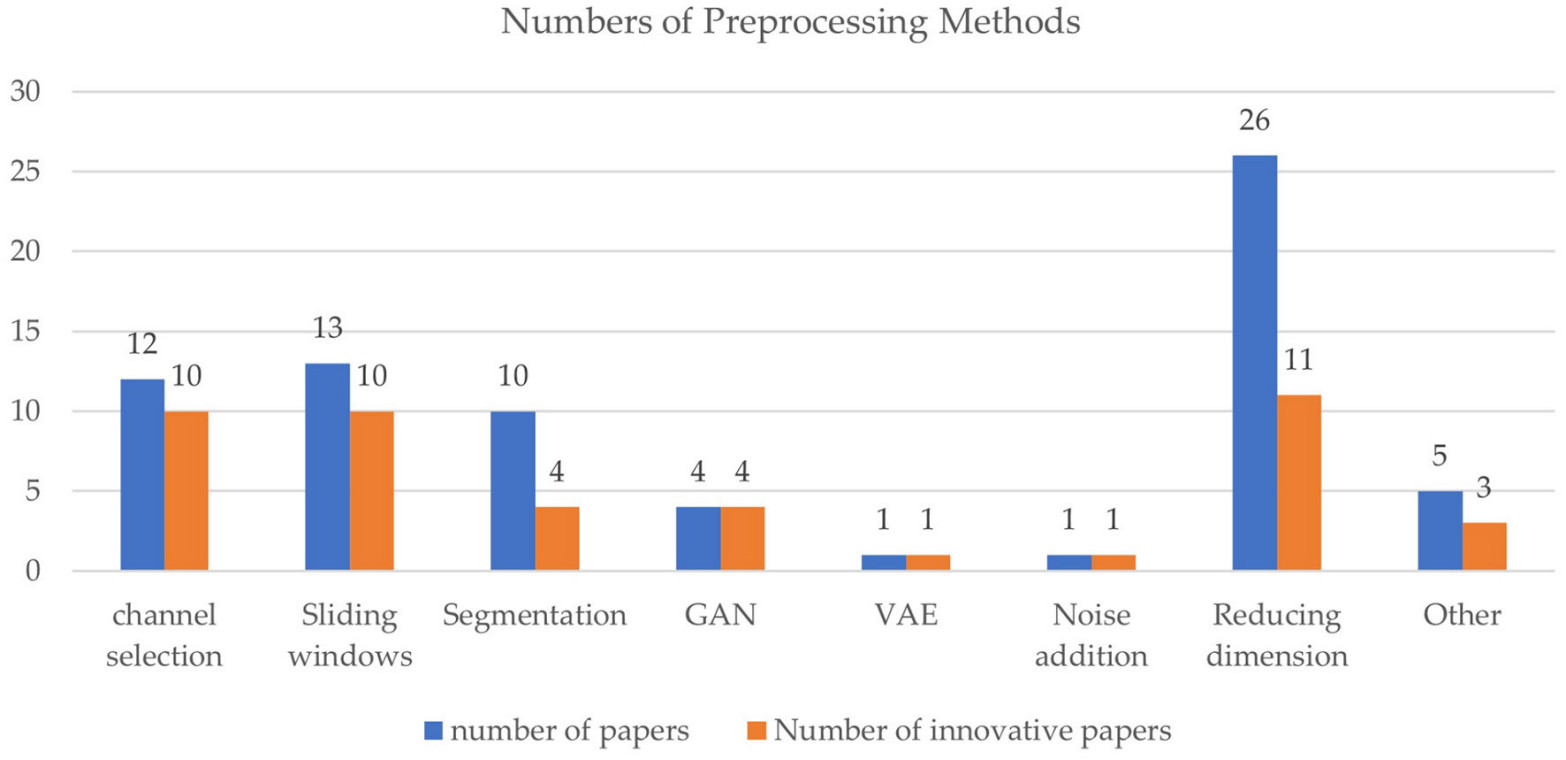
Comparison of preprocessing methods. Blue bars denote the number of papers involving a method, and the orange bars denote modified or new methods proposed.

In the 35 studies about preprocessing, innovative preprocessing methods include channel selection (10 papers, 28.6%), dimension reduction (11 papers, 31.4%), data augmentation (16 papers, 45.7%), etc., as shown in Figure 4. Dimension reduction is commonly used to reduce computation complexity, but only around half of the studies applying dimension reduction have innovative methods. While channel selection and sliding windows are also widely used, many papers proposed new channel selection or sliding window approaches.

According to Alzahab et al. (2021), among studies on hybrid Deep Learning (hDL) from 2015 to 2020, 21.28% did not apply any preprocessing step, 61.7% applied basic preprocessing such as bandpass filtering, and 17.02% applied more advanced preprocessing methods such as ICA and PCA. Our study shows that since 2021, 24 (100%) and 15 (62.5%) of the studies applying hDL performed preprocessing and advanced preprocessing methods, respectively. In addition, Alzahab et al. (2021) also pointed out that since none of the papers they reviewed compared performance between the presence and the absence of preprocessing, it cannot be confirmed whether preprocessing can improve accuracy. However, we found that several papers since 2021 have conducted comparative experiments on preprocessing and have clearly concluded that appropriate preprocessing methods can improve accuracy performance. For example, Lashgari et al. (2021) showed that by selecting the optimal channel (ACC = 81.73%) compared to no channel (ACC = 71.47%), the accuracy of the hybrid CNN and GRU algorithm was improved by 10.26%. Therefore, it has been proved that appropriate preprocessing methods can improve the performance of the entire processing task.

In the following section, we will discuss several directions of preprocessing research, including innovative preprocessing methods, channel selection, and data augmentation (including sliding windows, segmentation and recombination, noise injection, GAN, and VAE).

#### 3.2.1. Innovative preprocessing methods

The number of studies on improving classifier performance through preprocessing has increased significantly, with 60 (98.4%) of the 61papers including preprocessing, and 35 (57.4%) of them improving performance through innovative preprocessing approaches.

**Figure 5:**
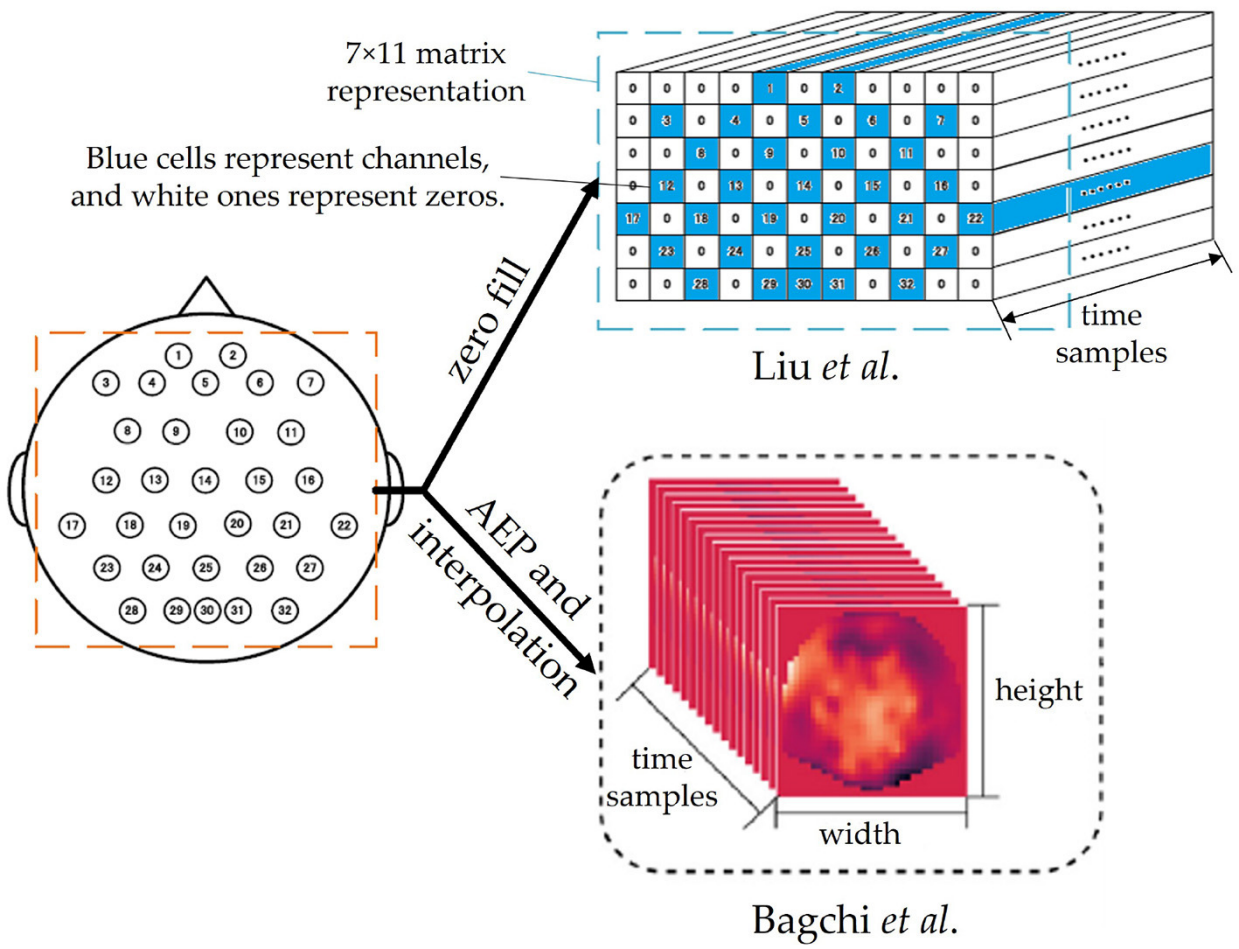
Two 3D representation methods of EEG signals in Liu and Yang (2021) and Bagchi and Bathula (2022), respectively. Both of them are in the shapes of width × height × time samples. The figure are partly cropped and modified from these two articles.

Many innovative preprocessing methods are related to the structure of deep learning networks. For example, Liu and Yang (2021) and Bagchi and Bathula (2022) both transform raw signals into 3D tensors, as shown in Figure 5. The former simply represents the positions of electrodes in a matrix roughly and fills zeros for the cells without electrodes, while the latter applies Azimuthal Equidistant Projection (AEP, a method to project a globe onto a plane) to transform the distribution of 3D electrodes into a 2D heatmap image and keep the relative distance of electrodes. After AEP and interpolation, the EEG signals become a video-like stream with plenty of 2D thermodynamic images and can be analyzed by ConvTransformer, which will be discussed in Section 3.3.1.

Autoencoder can extract the features and reduce the dimension of raw EEG signals by transforming them into a small vector. In Ayoobi and Sadeghian (2022), an LSTM-AutoEncoder is trained unsupervisedly, and the latent variables are used as the input of feature extraction algorithms. This shows that AE can extract valid features and greatly reduce the size of input signals.

#### 3.2.2. Selecting optimal channels

Among the 35 papers focused on preprocessing, 12 (34.3%) addressed channel selection, indicating that selecting optimal channels is a promising direction for improving classification performance. In the study conducted by Liu and Jin (2021), channel selection using the proposed Bispectrum and Euclidean Distance-based Channel Selection (BECS) algorithm resulted in significant improvements in classification accuracy for 18 out of 35 subjects (paired *t*-test, *p* < 0.05). The accuracy and ITR increased by 7.38% and 18.4%, respectively. However, performance did not significantly change for 10 subjects, and seven subjects experienced a decrease in accuracy. They also supposed that by ordering all the leads and fusing them with a fuzzy system, it is possible to automatically determine whether to select channels, thus avoiding performance degradation. Yin et al. (2021) using a voting approach to select optimal channels, which not only significantly improved the classification rate (*p* < 0.01) when compared with the traditional FBCSP algorithm, but also reduced computing complexity.

#### 3.2.3. Data augmentation

In our study, we reviewed 22 studies that applied data augmentation techniques to enhance EEG signal processing. Among them, we found that 16 studies adopted innovative data augmentation methods, which can be classified into several categories: sliding window (10 papers, 45.5%), segmentation and recombination (4 papers, 18.2%), Gaussian noise (one paper, 4.6%), GAN (4 papers, 18.2%), and VAE (one paper, 4.6%). Since a study may use multiple data augmentation methods, the total percentage exceeds 100%.

Many studies have adopted conventional data augmentation methods, including sliding windows and segmentation and recombination. For instance, Gaur et al. (2021) employed SW-Mode technology based on sliding window to reduce the differences among subjects, achieving superior performance compared to the best available technology in the dataset of stroke patients (ACC = 80%, *p* < 0.05). In another study by Lian et al. (2021), time windows were divided into 1 s to increase the number of training samples and satisfy the requirements of CNN, enhancing the stability and reducing the impact of individual differences (kappa = 0.78). Similarly, in a study by Islam et al. (2022), the dataset was divided into three different short decision windows (1, 2, and 3 s). They concluded that choosing a shorter decision window can reduce computational complexity, minimize the use of additional functionality for a single decision, and make the system faster. In yet another study by Ayoobi and Sadeghian (2022), the preprocessing step involved comparing fragments of various time windows, where the authors found that by clipping into short segments to match up self-attention mechanisms, the average classification accuracy increased by 13.9%, and computation complexity was reduced.

GAN can imitate the samples in the dataset and generate new EEG samples to improve the accuracy (Lashgari et al., 2021). However, poor training stability is a problem. For example, Song et al. (2022) used Auxiliary Classifier GAN (ACGAN), a variant of GAN, to generate new data to expand the training dataset, which met the requirements of deep learning and increased the accuracy by 1.7%.

Research has also explored diffusion models. Diffusion Model (DM), as an up-to-date substitute for GAN, can generate high-quality images and has a wide range of applications in AI painting. Thus, it is believed that diffusion models can also generate verisimilar EEG signals. For example, Duan et al. (2023) used diffusion to remove artifacts and improve cross-subject accuracy. However, there are few papers about the diffusion model in EEG signal generation, and more research is needed.

However, data augmentation also has some limitations which must be considered in its application. Here we list three main factors.

First, generating too much data through data augmentation is not appropriate. Beyond a certain amount, generating more data into the training dataset only increases the training time and will not improve the generalization of the model. For example, in the study by Lashgari et al. (2021), the model achieved the best accuracy (93.6%) after applying 15 times data augmentation on BCI Competition IV dataset 2a. If 20 times augmentation is adopted, the accuracy will decline instead.

Second, a major difference between synthetic EEG signals and images is that EEG signals cannot be directly interpreted. While GANs and other deep learning-based generators have demonstrated success in synthetic image generation, it is challenging to interpret the differences between real and synthetic EEG signals. Sliding windows and noise injection methods ensure that the augmented data are similar to real EEG signals, but GANs and Variational Autoencoders (VAEs) are less transparent, resulting in a new “black box”.

Third, selecting appropriate data augmentation methods in a given situation is crucial as different methods have both advantages and disadvantages. For example, sliding windows and segmentation and recombination can directly augment data in the input space, which is intuitive and has a low calculation cost (He et al., 2021). However, this method also increases the similarity of training data, which may cause overfitting and reduce the classification accuracy of the model.

### 3.3. Deep learning algorithms

As is mentioned in Section 1.3.2, CNN can extract temporal and spatial kernels by setting different sizes of kernels, and RNN can extract long-term temporal kernels. In our study, we find that most of the papers about DL use CNN alone or with other structures to extract features and use FC as the last layer to sort extracted features into given categories. Some papers also add an RNN layer after CNN. Also, batch normalization (BN) and dropout layers are applied widely to avoid overfitting.

**Figure 6:**
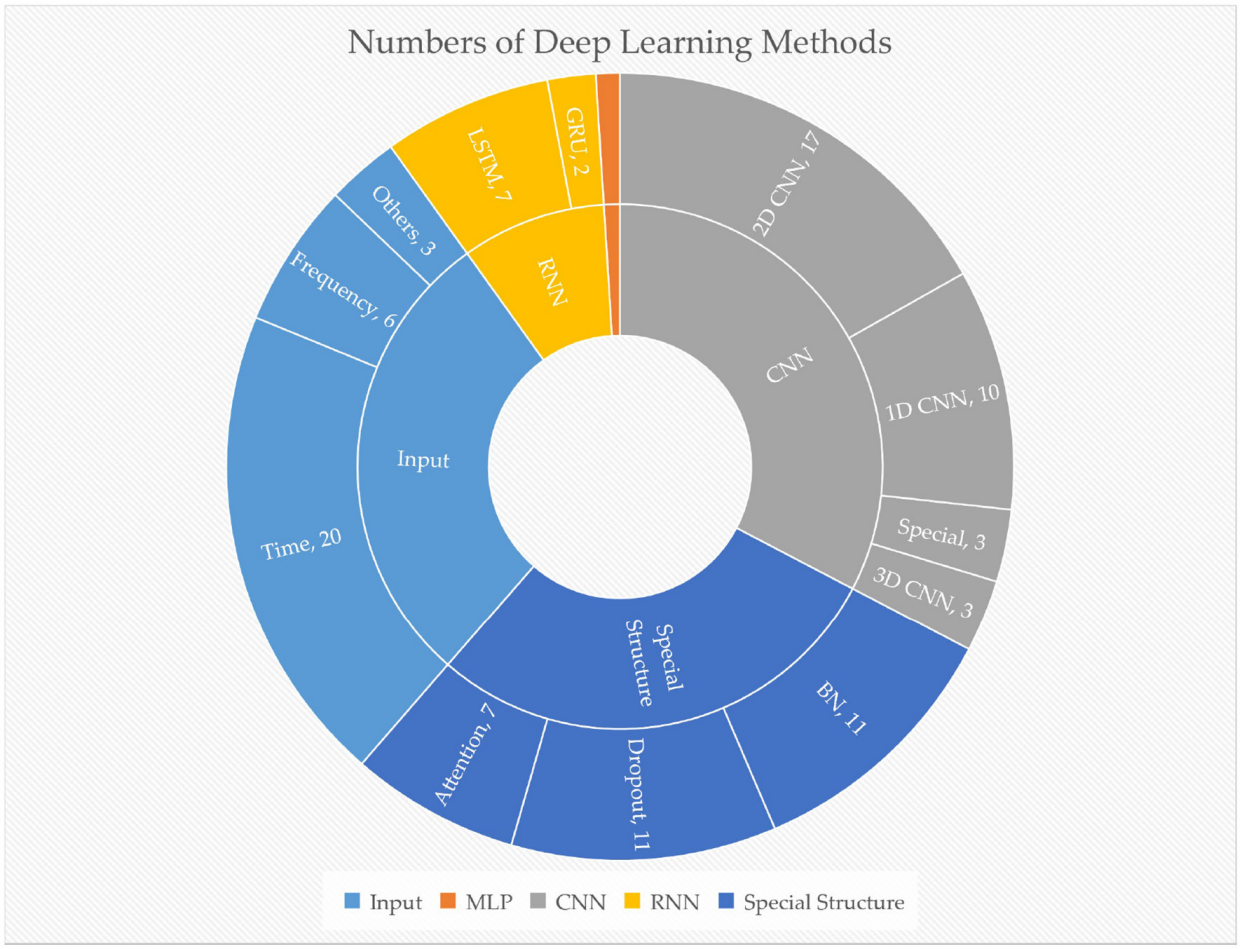
The number of different deep learning methods used in reviewed papers. The orange sector represents MLP, with one paper. Special CNN includes graph CNN, etc.

Among the papers reviewed in our study, 28 studies used DL algorithms, with the majority relying on CNNs (26 papers, 92.9%), followed by RNNs (eight papers, 28.6%) and MLP (one paper, 3.6%). Most of the CNN and RNN algorithms proposed innovative methods. Notably, since 2021, CNN-based algorithms have accounted for 42.6% of the 61reviewed papers, indicating that CNN is the mainstream classification and DL approach for EEG signal processing. Out of the 28DL papers reviewed, 20 studies used time-domain signals as input, while six studies used time-frequency domain signals. The number of different methods or structures is depicted in Figure 6. As some studies used multiple methods, the sum of each sector may exceed the total number.

Our research indicates that the prevalence of DL and the popularity of CNNs as a classification algorithm can be attributed to three factors:

1. DL can be applied to not only the spatial and time domains but also the frequency and time-frequency domains. CNNs are predominantly used to extract spatial features, while RNNs can extract longer temporal features than CNNs.
2. Classification accuracy is highly dependent on the amount of training data, and limited training data often leads to low accuracy. Data augmentation and transfer learning have partially resolved this issue in recent years. Data augmentation expands the amount of data by applying various preprocessing techniques, while transfer learning leverages knowledge and experience from other fields to train a model with a smaller dataset. Transfer learning shortens the training time and is less affected by individual differences.
3. DL algorithms reduce the computational burden and enhance BCI performance by sharing parameters or constructing a shallow neural network that is consistent with the characteristics of EEG.

#### 3.3.1. Application and innovation in CNN

CNNs are capable of extracting both spatial and short-term temporal features using 1 × *M* and *N* × 1 kernels, respectively, when the input has the shape *C* × *T*. Research on CNNs can be classified into three categories: (1) improving proposed networks, (2) modifying networks from other domains, and (3) proposing innovative network structures.

First, to improve proposed networks. As mentioned in Section 1.3.2, EEGNet is a network designed specifically according to the characteristics of EEG signals (Lawhern et al., 2018). Various modifications have been made to EEGNet to improve performance. For example, Li H. et al. (2022) added an FC layer to concatenate the output of three convolution layers to aggregate different features and improve the accuracy of EEGNet. Vega et al. (2021) added a Fuzzy Neural Block (FNB) after EEGNet and demonstrated that FNB can slightly enhance the accuracy of subject-dependent classification.

Second, to adapt the networks from other realms. Researchers have applied innovative structures from the fields of imaging processing and computer vision to EEG signal processing. For instance, Bagchi and Bathula (2022) modified the ConvTransformer network, originally designed for video processing, to process EEG signals and achieve the highest accuracy among several methods. Here, the raw EEG signals are preprocessed into a video-like stream, which serves as the input of ConvTransformer, similar to the approach used in video processing. However, the ConvTransformer model has a large computational complexity due to a large number of parameters. In addition, Lin et al. (2022) inserted the Spatial Attention Mechanism (SAM) from the field of imaging processing into their network to extract the salient frequency of EEG signals.

Third, to propose an innovative network structure. Compared to traditional 2D-CNN, various methods of deep learning have been developed. Mattioli et al. (2022) proposed a 1D CNN, a special convolutional layer that accepts input of shape *T* × *C* and uses *Q* × *C*-shaped kernels to process the input, thereby squeezing the second dimension and outputting a tensor of shape *T* × *O*, where *O* is the number of output channels. Multi 1D CNNs were constructed sequentially, which reduces computational cost while achieving high accuracy. Lashgari et al. (2021) proposed a relatively simple network that uses a shared-parameter convolutional layer as sliding windows to process signals without any conventional preprocessing methods. They then employ CNN and self-attention mechanisms to extract features and an FC layer for classification. The network achieved the highest accuracy compared to other state-of-the-art networks, mainly because it abandoned prior methods such as preprocessing and manual hyperparameter selection, allowing all parameters to be trained and updated automatically.

There are also deep learning studies focusing on particular applications of EEG-BCI. For instance, aiming to develop a BCI for people with communication disabilities to control the movement of a device, Vorontsova et al. (2021) designed a simple network using ResNet and GRU but without the last FC layer to classify the EEG signals into correct words during silent speech. They hypothesized that a smaller dataset on one subject (i.e., subject-dependent dataset) will contribute to higher accuracy, which was demonstrated by their experiment results. Similarly, Vega et al. (2021) applied P300-based EEG signals on controlling smart appliances. They collected EEG signals from both healthy and post-stroke subjects and designed the aforementioned EEGNet with FNB to classify these data. The performance of these applications has shown the effectiveness and prospect of deep learning-based EEG signal processing methods and the possibility of real-life BCI devices.

#### 3.3.2. Multi-network structure fusion

There exist various neural networks and structures, such as CNN, RNN, attention mechanisms, and AutoEncoder (Encoder-Decoder) structures, each with different performance in various application scenarios. The fusion of multiple structures can improve classification accuracy by combining different features (Singh et al., 2021). It has been observed that many studies apply dropout, BN, and attention mechanisms to deep learning algorithms. Among the 28papers surveyed, 11 (39.3%) used dropout, 11 (39.3%) used BN, and 7 (25%) used attention mechanisms.

Lin et al. (2022) proposed Phase Learning and Frequency Attention Network (PLFA-Net), which combines a phase-learning module, SAM, feature-extracting CNN, and a fully connected layer. The phase-learning module calculates the linear combination of the real and imaginary parts of FFT outputs to learn phase information. The SAM extracts frequency features as mentioned above. The feature-extracting CNN, a conventional convolutional layer, extracts features of both time and channels. PLFA-Net performs better than CCA in high frequency but worse in low frequency, probably because it extracts low-amplitude information of high frequency well but is affected by low-frequency noise.

Lian et al. (2021) combined a Shallow CNN (SCNN), BiLSTM, and attention mechanisms to improve EEG classification. The design of SCNN is inspired by Visual Geometry Group (VGG), but SCNN uses rectangular kernels with shapes of 1 × 5 and 1 × 3 instead of square kernels to extract temporal features. Attention mechanisms are used after BiLSTM to fuse the features of SCNN and BiLSTM.

Li and Sun (2022) used modified EEGNet and ConvLSTM to process EEG signals. ConvLSTM uses convolution operations to pass the hidden state, combining both RNN and CNN. Attention mechanisms are also adopted before the input of ConvLSTM. This model achieved better results on several datasets.

#### 3.3.3. Problems and future directions of deep learning

Although deep learning has been shown to be effective in processing EEG signals, several notable problems still exist, which also serve as future directions for research in EEG signal processing with deep learning. In the following sections, we highlight some of these issues and provide potential avenues for addressing them.

**Figure 7:**
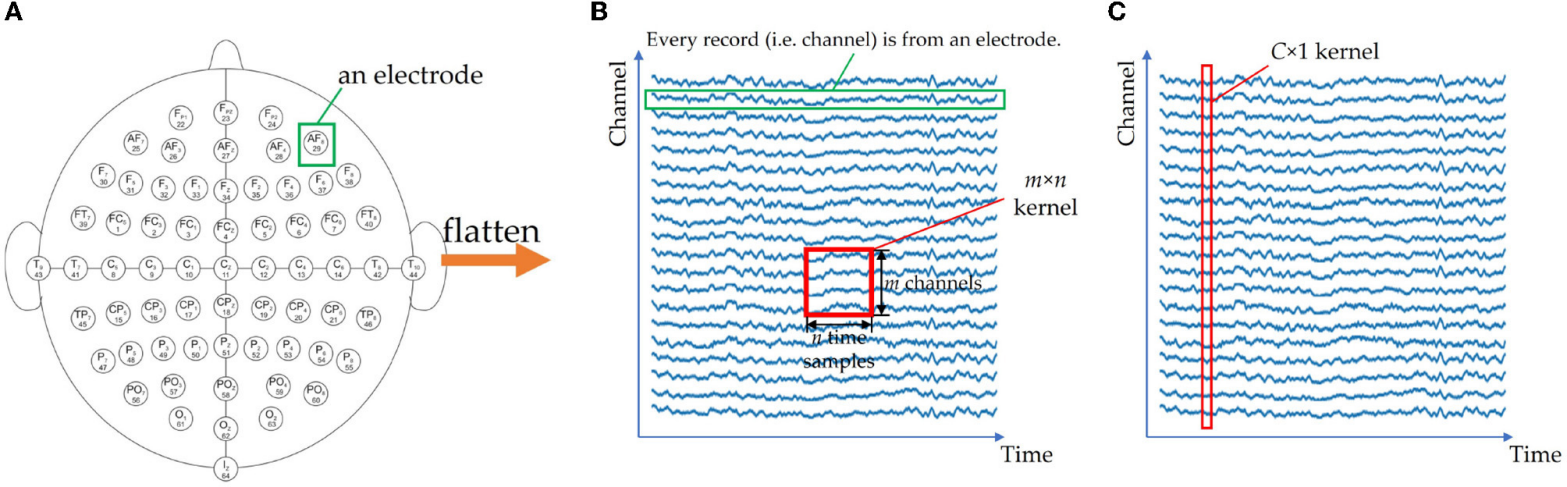
If 2D electrodes **(A)** are squeezed into a 1D channel arrangement **(B)**, the spatial relationship of electrodes is lost. An *m* × *n* CNN kernel cannot extract the information fully, since it only extracts *n* channels every time. For EEGNet **(C)**, a *C* × 1 kernel is used to extract the features from all channels. **(A)** 2D sphere of collecting device. **(B)** 1D representation and a normal CNN kernel. **(C)** *C* × 1 kernel of EEGNet.

The first problem is related to the shape of the input. While CNNs typically accept 2D images or 3D multi-channel images as input, EEG signals are multi-channel 1D sequences with a shape of *C* × *T*. Directly using a 2D *C* × *T* matrix as input can lead to insufficient feature extraction during convolutional operations due to the electrodes being adjacent to four surrounding electrodes on a 3D sphere, as opposed to only two adjacent electrodes in the matrix. Figure 7 illustrates this issue. Some researchers ignore the input shape and properties of CNNs, using inappropriate inputs and conventional 2D convolutional layers, which can result in poor correlation extraction. For example, in Islam et al. (2022), after applying wavelet transform as a feature extraction step, the spectrograms of all channels are concatenated into a large 2D image, which may extract redundant features due to the lack of apparent relations between the border of the image. To overcome these issues, researchers have proposed innovative solutions such as considering each channel as an independent sample, transforming the signals into 3D tensors to preserve the relative positions of electrodes (Liu and Yang, 2021; Bagchi and Bathula, 2022), or using a *C* × 1 kernel for depthwise convolutional layers in models such as EEGNet (Lawhern et al., 2018) and EEG-TCFNet (Vega et al., 2021). In the future, further attention is needed in designing CNN structures that can accommodate the shape and characteristics of EEG signals to effectively extract features.

Another problem concerns the accuracy of cross-subject tasks, where many studies have shown high accuracy in within-subject tasks but lower accuracy in cross-subject tasks. For instance, in the study of Mattioli et al. (2022), the accuracy drops sharply from 99% in within-subject tasks to approximately 50% when applying transfer learning to other subjects, indicating that the parameters overfit the given individuals and cannot be generalized easily to new individuals. This issue presents a bottleneck for EEG applications and BCI technology, where a universal classifier for all participants is yet to be designed. In fact, Singh et al. (2021) have summarized some methods to improve subject-independent accuracy, but most of them are non-deep learning methods or fusions of non-DL and DL methods. Further research on deep learning-based EEG signal processing to address this problem and improve cross-subject accuracy is still needed.

The balance between accuracy and time cost is another challenge that requires consideration in many cases. Deeper and more complex networks often lead to higher accuracy but also longer computing time, which can be problematic in real-time EEG-based applications like BCI for controlling devices. Some studies, such as the study of Bagchi and Bathula (2022), pursue higher accuracy but achieve it with a large number of parameters and high time cost. On the other hand, some studies have used special structures like separable convolutional layers, which can maintain high accuracy without significantly increasing computing time. In the future, achieving a balance between accuracy and computing time will remain an important consideration.

Finally, the lack of interpretability in both EEG and deep learning hinders further development. As the functions of our brains are still not fully understood, interpreting EEG signals and understanding the workings of deep learning models can be challenging. Deep learning is often considered a “black box” (Adadi and Berrada, 2018), which can make it difficult to explain and understand EEG DL models. Additionally, the lack of interpretability is also a factor contributing to the low cross-subject accuracy, as we do not know the specific variations in EEG signals between different individuals. Many papers attempt to explain the reason why their networks are efficient in terms of network structure, but the existing reasons are still very subjective. Furthermore, Vallabhaneni et al. (2021) stated that pathological mechanisms are more important than classification accuracy in medical and psychological applications of EEG and BCI. But from the papers we reviewed, researchers are still mainly paying attention to high performance, or more precisely, high accuracy. Hence in medical realms, deep learning of EEG cannot still be put into practical applications, leaving a significant unsolved problem.

### 3.4. Fusion of different methods

Currently, there exist numerous classification algorithms. However, their performance varies significantly across different paradigms and application scenarios. The *No Free Lunch* Theorem asserts that it is impossible to find a single algorithm that generalizes well on any distribution of data (Wolpert and Macready, 1997). Additionally, many algorithms encounter challenges during processing, such as insufficient feature extraction, overlooking global network characteristics, and inability to identify the physical function of the brain. Furthermore, as preprocessing methods and deep learning algorithms develop and merge, the partitions between preprocessing, feature extraction, and classification become blurred. In the review conducted by Saeidi et al. (2021), ICA and PCA are classified into both preprocessing and feature extraction methods. In most deep learning studies, features are considered automatically extracted by neural networks, so there is not a single step of feature extraction. Multi-algorithm fusion, as opposed to a single algorithm, can optimize feature selection, decrease computational complexity, and improve classification accuracy (Singh et al., 2021).

**Figure 8:**
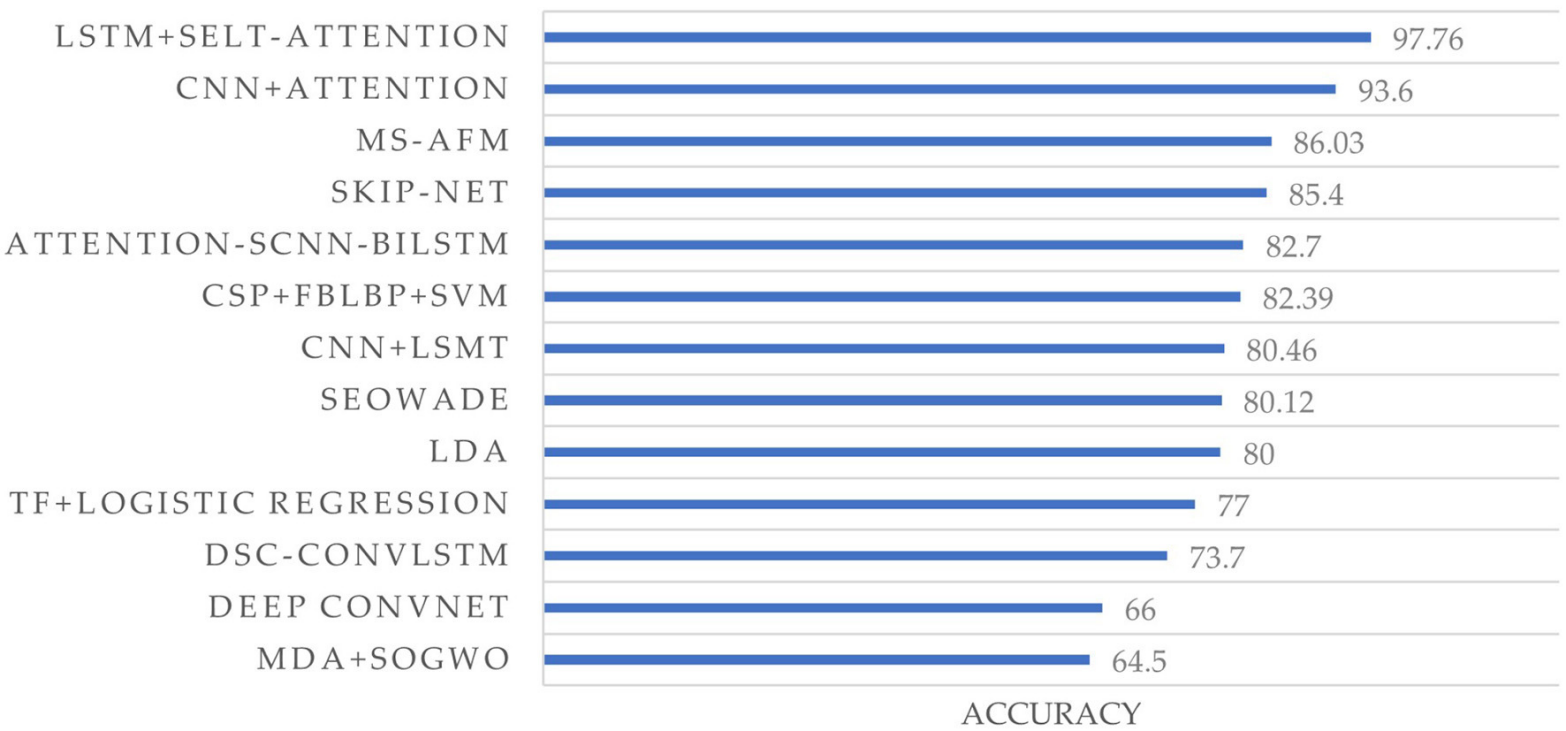
Accuracy comparison of different methods on the BCI competition IV dataset 2a. Each label on the vertical axis represents a method in a study, which is from Gaur et al. (2021); Lashgari et al. (2021); Lian et al. (2021); Liu and Yang (2021); Liu et al. (2021); Qi et al. (2021); Ali et al. (2022); Ayoobi and Sadeghian (2022); Chang et al. (2022); Chen L. et al. (2022); Ko et al. (2022); Li and Sun (2022); Li H. et al. (2022), and Tang et al. (2022), from top to bottom, respectively.

Our survey reveals that 45 studies (73.8%) employed the fusion of more than two methods for feature extraction and classification, leading to a significant improvement in performance. Multi-algorithm fusion is becoming a new trend in EEG signal processing. Additionally, 14 papers utilized the BCI Competition IV 2a dataset for their studies, as shown in Figure 8. Liu et al. (2021) utilized the fusion of LSTM and self-attention and achieved the highest accuracy (97.7%) among 14 papers.

#### 3.4.1. Multi-conventional method fusion

**Figure 9:**
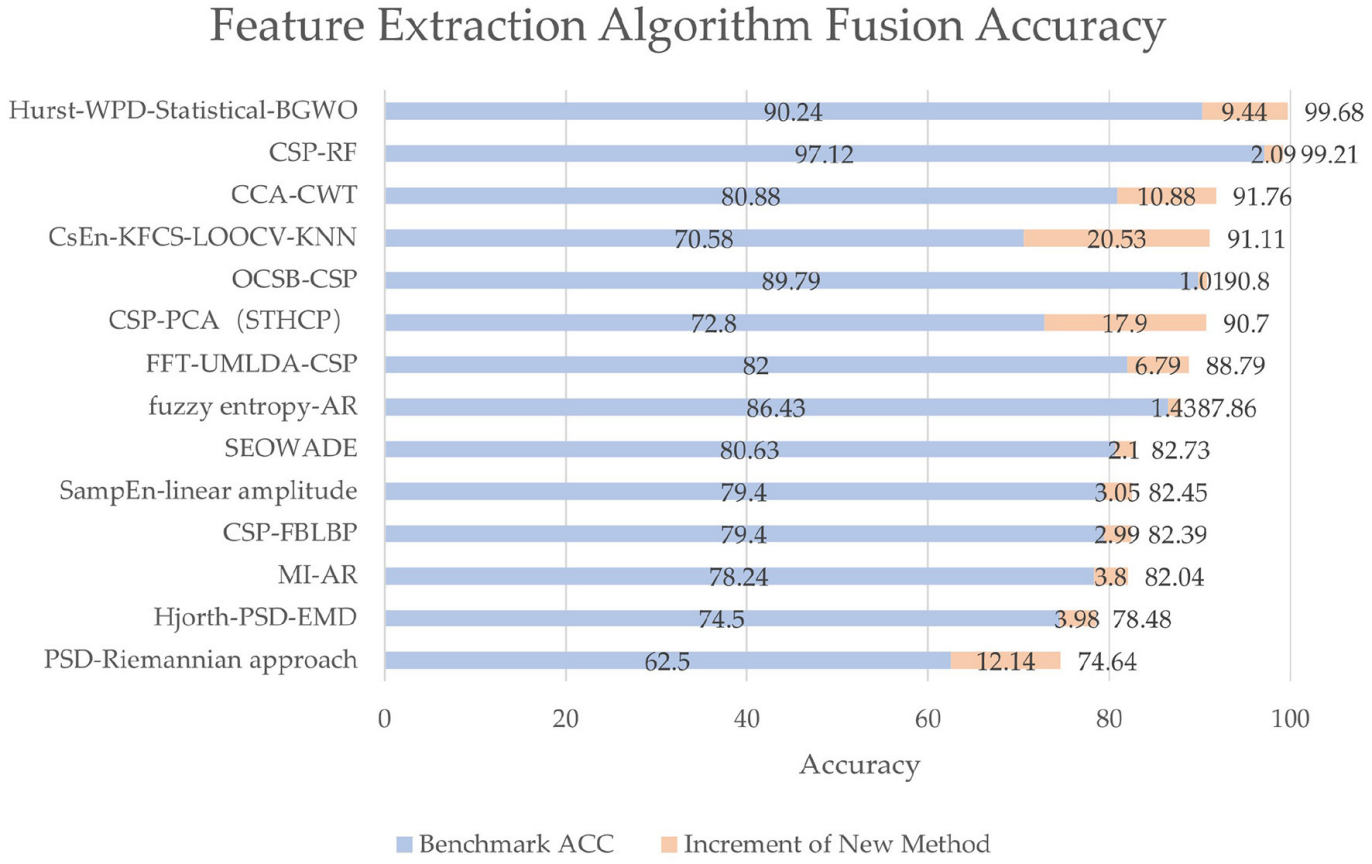
The accuracy and accuracy increment of different fusion methods. The benchmark algorithms are often methods in previous studies or conventional algorithms like simply CSP, whose accuracy is denoted by blue bars. The innovative methods mean new methods proposed in the papers, whose accuracy increments are denoted by orange bars. Each label on the vertical axis represents a method in a study, which is from Du et al. (2021); Gao N. et al. (2021); Qi et al. (2021); Rashid et al. (2021); Wang and Quan (2021); Xu C. et al. (2021); Yin et al. (2021); Zhang Y. et al. (2021); Algarni et al. (2022); Cui et al. (2022); Jia et al. (2022); Ma et al. (2022); Pei et al. (2022), and Tang et al. (2022), from top to bottom, respectively.

Despite the prevalence of deep learning, many conventional feature extraction methods are still applied to EEG signals, especially in feature extraction. In practice, when choosing a variety of conventional algorithms, it is necessary to consider the influence of various factors such as the number of samples that can be obtained, training time, and test methods. Through combination, the optimal feature-extracting ability can be obtained, and the time cost of training and classification can be saved, along with good generalization ability. Our research shows that 16 papers (26.2%) using feature extraction algorithms involve feature extraction fusion algorithms, indicating that feature extraction algorithms also have a trend of fusion development. The accuracy and accuracy increment are shown in Figure 9. Although different benchmarks or classification algorithms are used among different studies, they all achieve at least 1% accuracy increments.

For example, Sun et al. (2021) proposed the Discriminative Canonical Pattern Matching (DCPM) algorithm, which integrates DSP, CCA, and pattern matching, and achieved the best performance in various situations. Compared with LDA-related algorithms, DCPM performs better when the number of training samples is small or the number of features is too large. Compared with SVM-related algorithms, DCPM avoids the long-term parameter selection process during use, making the application process easier.

Ma et al. (2022) proposed the CCA-CWT-SVM fusion algorithm, which combines the features extracted by CCA with CWT, to achieve feature complementarity, and thus significantly improves the target accuracy within a limited time.

Yin et al. (2021) proposed a channel-based optimal sparse time-frequency block common space pattern (OCSB-CSP) feature extraction method to improve model classification accuracy and computational efficiency. A channel selection method based on Pearson correlation coefficients is first invoked to reduce the redundant information between channels and to mark the best channel for subsequent processing. Then, the discriminative ability of each time-frequency block is measured by defining the Fisher ratio index to sparse the time-frequency blocks, which significantly reduces the data dimensionality, and the selected time-frequency blocks are mostly distributed in the frequency bands related to the MI task. Finally, the p-shape analysis of Lasso regression is performed to select the extracted multi-block CSP features and use SVM for classification. The results show that the proposed OCSB-CSP algorithm achieves higher classification accuracy while reducing the computational burden of the model.

#### 3.4.2. Fusion of conventional methods and deep learning

In addition to modifying deep learning algorithms as discussed in Section 3.3.1, some studies have explored the fusion of conventional methods and deep learning. In this section, we review some innovative fusions of both conventional methods and deep learning.

Islam et al. (2022) proposed a fusion of CNN and KNN. The features extracted by CNN are treated as 1D vectors in ℝ^*m*^ and classified by KNN using Euclidean distance.

Algarni et al. (2022) proposed a fusion of Hurst index, WPD, statistical features, Binary Gray Wolf Optimization (BGWO) algorithm, and BiLSTM. Hurst index is used to measure the long-term memory changes of time series, WPD is used to better filter the discrete-time signal, and statistical features are used to analyze time-domain features. The fusion of Hurst, WPD, and statistical features is used to extract features. BGWO is applied to select features and eliminate redundant features while retaining important information. BiLSTM is then used to extract features further, and finally, an FC layer is used for classification.

#### 3.4.3. Future directions of multi-method fusion

Multi-method fusion is a promising trend in EEG signal processing. We will attempt to give some reasons why multi-method fusion is important and summarize the tendencies of multi-method fusion in this section.

First, the characteristics of the brain have not been fully explored. EEG comes from the neural activities in our brain, and the aim of EEG signal processing is to decode and obtain information from our brain. However, nowadays there are few researchers who adopt machine learning fusion focusing on the explanations of the effectiveness of algorithms, as well as associating the algorithms with cognitive mechanisms. While the effectiveness of fusion methods can be indirectly demonstrated through comparing performance like accuracy, what feature each method exactly extracts, and what the neurologic meanings of features are, are not clear, so the cognitive mechanisms behind the fusion methods are not fully understood. Thus, the optimal solutions or a general explanation of algorithm selection have not been proposed (Chen J. et al., 2022).

Second, in addition to algorithm fusion, other fusion approaches such as multi-sample fusion are also worth exploring. Fan et al. (2021) used multi-sample fusion to classify EEG signals using SVM, where multiple samples from the same experimental conditions were combined to improve classification accuracy. This significantly improved the accuracy of SVM classification than that of a single sample.

Third, a single feature may not effectively capture the physiological behavior of the brain. Future studies may introduce more parameters to improve classification accuracy. However, method fusion may lead to increased time and space complexity, resulting in longer training and prediction times, which limits the practical application of the model (Lu et al., 2022). Therefore, it is important to balance the parameter number and time cost to avoid overfitting and excessive time and space complexity. For example, in Section 3.4.2, we discussed the study of Algarni et al. (2022) that used multi-feature extraction stages and BiLSTM to reduce the dimensionality of data and improve classification accuracy. BiLSTM reduces the high dimensionality of data, which reduces complexity and leads to less classification time and improved performance.

In summary, multi-method fusion is a promising direction for EEG signal processing, and more research is needed to fully explore its potential. Future studies may focus on understanding the cognitive mechanisms behind the fusion methods, exploring other fusion approaches, and reducing the limitations and complexity of the model.

## 4. Conclusions

In this paper, we have reviewed 61studies of EEG signal processing. We have discussed different preprocessing methods and highlighted the effectiveness of proper preprocessing methods to increase accuracy, which solves the problems in the review of Alzahab et al. (2021). Furthermore, we have observed the wide adoption of deep learning methods in EEG signal processing and discussed some reasons why they have become prevalent. We have also noted that many studies apply multi-method fusion, using both conventional algorithms and deep learning. This summarization can show some future directions to the researchers focusing on EEG signal processing.

Despite these advancements, we still face significant challenges in EEG-based BCI systems and EEG signal processing due to our limited understanding of the brain. The problem of low cross-subject accuracy also remains unsolved, indicating a limited generalization ability. Designing a more robust system with stronger generalization ability and less time cost remains an open question for future research.

## Author contributions

CS and CM conceived the ideas and designed the study. CS collected the relevant literature and wrote the first draft of the manuscript. CM provided writing supervision and critical revisions and finalized the manuscript. Both authors contributed to the article and approved the submitted version.
