# Supplementary material for: Survey on the research direction of EEG-based signal processing
Source: Front Neurosci. 2023 Jul 13;17:1203059. doi: 10.3389/fnins.2023.1203059 (PMC10372445; doi:10.3389/fnins.2023.1203059)
Supplement: Supplementary file 1 [file Table_1.pdf]

Table 1: The methods of reviewed paper

Ref.	Month and Year	Paradigm	Dataset	Pre-processing	Feature Extraction	Classification Algorithms	Indicators	Main Direction
[1]	Jan 2021	MI	IV 2a, Stroke patient dataset	Filtering, segmentation, sliding window	CSP	LDA	ACC *approx* 80%, kappa = 0.6	sliding window
[2]	Jan 2021		III 4a, III 3a, IV 2a	Filtering, time windows	SEOWADE		ACC: III 4a 82.43, III 3a 90.65, IV 2a 80.12	Feature extraction fusion
[3]	Jan 2021	MI	III 3a, IV 1	Granger causality, channel selection	regularized CSP	linear SVM	ACC: 93.03 – 96.98%	channel selection
[4]	Jan 2021	MI	Experimental dataset, 11 subjects	Filtering, downsample		EEGNet + TL	ACC: avg 66.36%	Deep learning algorithm fusion
[5]	Jan 2021	simulated driving	Emergency braking intent detection in 7 subjects	ICA	CsEn + KFCS + LOOCV + KNN	SVM	ACC: avg. 83%	Feature extraction fusion
[6]	Feb 2021	MI	IV 2a	Filtering, time window, AE		Attention + CNN + BiLSTM	ACC 82.7%, kappa 0.78	Data augmentation, deep learning algorithm fusion
[7]	Mar 2021		IV 2b	Taguchi method + GRA	Hjorth	SVM	ACC: 82.58%	preprocessing innovation
[8]	Mar 2021	MI	III 1, III 3a, III 4a, IV 2	Filtering, downsample	CSP + Random Forest	Random subspace ERS + KNN	acc: III 1 99.21%, III 3a 93.19%, III 4a 93.57%, IV 2 90.32%	Feature extraction fusion, Classification algorithm fusion
[9]	May 2021	MI	IV 2b	Filtering, downsampling	Hjorth parameters + statistics methods + EMD	hybrid TL + SVM	ACC: 79.4% – 87.5% from the target domain, 78.48% for hybrid	Feature extraction fusion, algorithm fusion
[10]	May 2021		WAY-EEG-GAL dataset, IV 2a	Bandpass filtering, Class balancing cropping policy, 3D sliding window		Three branches 3D CNN	WAY-EEG-GAL,66.9 – 91.8, IV-2a 64.5	Preprocessing, deep learning

Table 1: Cont.

Ref.	Month and Year	Paradigm	Dataset	Pre-processing	Feature Extraction	Classification Algorithms	Indicators	Main Direction
[11]	May 2021	MI	IV 1, III 4a	Select channel via GA	Sparse time-frequency block common space mode (OCSB-CSP), channel selection	RBF -SVM	IV.1 84.78%, III 4a 90.80%	channel selection, feature extraction fusion
[12]	May 2021	SSVEP	32 Subjects, Group A Acc > 70%, Group B Acc < 70%	Filtering, downsample	Chaos		Group A ACC 91.11%, ITR 170.67; Group B ACC 85.41%, ITR 152.40	
[13]	Jun 2021		SEED	sample selection		MCD-DA + TL	ACC: cross-participants 88.33%, cross-time 92.90%	deep learning algorithm fusion
[14]	Jun 2021	P300	III 1, III 2.	Baseline correction, Windsorzing, Local Binary Patterning.		Autoencoder + CNN	ACC: III 1 79.75% – 87.5%, III 2 75.1 – 82.71; avg. ITR: 16.83, 33 bits/min	Preprocessing, deep learning algorithm fusion
[15]	Jun 2021	SSVEP	Tsinghua standard dataset	BECS		CCA	ACC: 90.35%, ITR: 85.96 bit/min	Preprocessing channel selection
[16]	Jun 2021	ErrP	2015 Challenge Dataset, Horizon 2020 Program Data	Down-sampling, filtering		DCPM (DSP + CCA + pattern matching)	ACC: 76.59% and 77.10%	Algorithm fusion
[17]	Aug 2021	MI	experimental dataset, IV 2a, IV 2b	sliding window (1D CNN), channel selection, GAN		CNN + attention, TL	ACC: 93.6% for IV 2a, 87.83% for IV 2b	Preprocessing, Data augmentation, Deep learning algorithm fusion
[18]	Aug 2021	MI	IV 2a, experimental dataset	Reshape into 1D		1D CNN + GRU	ACC: 99.40% for IV 2a, 92.56% for experimental dataset	Innovative preprocessing, deep learning algorithm fusion
[19]	Sep 2021		Auditory dataset, visual datasets	ICA, filtering, sliding window		ResNet	ACC: Auditory 77%, visual 75%	Data augmentation
[20]	Sep 2021	SSVEP	own dataset	time slicing	PSD	Event-based ACA	ACC 93.47%	Algorithm fusion
[21]	Sep 2021	MI	EEG dataset: 109 subjects	(by EEGLAB)		Probabilistic Neural Network	ACC: 98.65%	Innovative classification algorithms

Table 1: Cont.

Ref.	Month and Year	Paradigm	Dataset	Pre-processing	Feature Extraction	Classification Algorithms	Indicators	Main Direction
[22]	Sep 2021	MI	IV 1		PSD + Riemann method	KNN	ACC: 74.64%	Feature extraction fusion
[23]	Sep 2021	MI	II 3, IV 2b	EMD	Fuzzy entropy + AR	SVM with SSSE	ACC: II.3 avg. 82.47%, IV.2b avg. 79.25%	preprocessing, feature extraction fusion, classification algorithms innovation
[24]	Nov 2021	SSVEP	BCI-SSVEP-Training Dataset	The Butterworth filter separates frequencies 12, 15, and 20 Hz	FBCSP	SVM	12 Hz 92.91, 15 Hz 98.95, 20 Hz 94.50, ITR 76.34 bit/min	preprocessing
[25]	Nov 2021	SSVEP and SS-MVEP	own dataset	multi methods by EEGLAB	limited traversal viewable	Broad Learning System	ACC: SSVEP 96.22%; SSMVEP 74.54%	Feature extraction
[26]	Nov 2021	MI	109 subject experimental datasets	Filtering, ICA + WT	CSP	BLDA (Bayesian Linear Discriminative Analysis)	87.42	preprocessing
[27]	Nov 2021	silent speech	own dataset	downsampling, PCA + t-SNE and ICA	FFT	ResNet18 + GRU	ACC: 84.5% with pseudo-words, avg. 88%	preprocessing, deep learning fusion
[28]	Nov 2021	MI	own dataset	The filter is divided into 6 bands: 0.3 s, 0.6 s, 1 s, 1.2 s, 1.5 s, 2 sliding windows	rCSP	KNN + voting.	ACC: 78.9% (2 s window).	Preprocessing, algorithm fusion
[29]	Dec 2021	P300	own dataset	filtering, time slicing, downsampling		Multi-sample fusion SVM	ACC: 99.5% after 4 rounds	Downsampling, multi-sample fusion
[30]	Dec 2021	MI	EEG Motor Movement / Imagery; Dataset V 1.0.0	EEGLAB, Semi-automatic ICA, channel selection	ERD_AB (Alpha and Beta band between 8 – 25 Hz)	SVM	94.75	Preprocess channel selection
[31]	Dec 2021		Make online datasets public	Filtering, downsample, Split 1 s, 2 s, 3 s three short decision windows	CWT	CKNN (CNN + KNN)	92.26	Preprocessing, deep learning algorithm fusion

Table 1: Cont.

Ref.	Month and Year	Paradigm	Dataset	Pre-processing	Feature Extraction	Classification Algorithms	Indicators	Main Direction
[32]	Dec 2021	MI	own dataset	Artifacts removal, baseline correction, filtering and channel selection	Sample entropy and linear amplitude	LDA and SVM	ACC: offline 80.75%, real-time 82.45%	feature extraction fusion
[33]	Jan 2022	MI	own dataset	Filtering	mutual information + AR	SVM	ACC: Avg 82.04	Feature extraction fusion
[34]	Jan 2022		fusion of EEG and EMG signals	multi methods by EEGLAB		CNN + TL	ACC: 81.35% – 87.49%	Deep learning algorithm fusion
[35]	Jan 2022	MI	IV 2a	filtering, sliding window		WMFF (Improved EEGNet), CMFF (CNN + LSMT)	76.19, 80.46	Deep learning algorithm fusion
[36]	Jan 2022	MI	own dataset	SMOTE		1D CNN + TL	ACC: 99.38% for groups, 50% for cross-subject and TL	Data augmentation; transfer learning; deep learning algorithm fusion
[37]	Jan 2022	VR video stimuli	own dataset	channel selection, filtering, interpolation	FFT	Multi methods	ACC: cross-subject 85.01%, single subject 97.66%	channel selection
[38]	Jan 2022	MI	IV 2a, 2b and own dataset	Filtering, time window	CSP-FBLBP	SVM	ACC: avg. 82.39%, IV.2a 83.55%, IV.2b 79.72%; other data 75.99%	Feature extraction fusion
[39]	Feb 2022	RSVP	own dataset	filtering, channel selection, segmentation	STHCP (CSP + PCA)	LDA	ACC 90.7%	Feature extraction fusion
[40]	Feb 2022	SSVEP	BETA public SSVEP dataset	filtering, time slicing	FFT	PLFA-Net (CNN + SAM)	ACC 81.21%, 83.17% after DA	data augmentation; deep learning algorithm fusion
[41]	Feb 2022	SSVEP	own dataset	(by EEGLAB)	CCA + WT	SVM	ACC: 91.76%, ITR 48.92 bit/min	Feature extraction fusion
[42]	Feb 2022	MI	III 4a, IV. 1	CSR - CS	CSP	SVM	III 4a 88.61%, IV 1 83.9%	channel selection

Table 1: Cont.

Ref.	Month and Year	Paradigm	Dataset	Pre-processing	Feature Extraction	Classification Algorithms	Indicators	Main Direction
[43]	Mar 2022	MI	II 3, IV 2b, IV 2a	Data Auga (Gradient Norm Adversarial Augmentation).	Anchored-STFT	Skip-Net	II-3 Acc 90.7, Kappa 0.814; IV-2b 89.5, 81.8,76.0IV-2a 85.4, 69.1, 80.9	data augmentation, innovative feature extraction, deep learning algorithms
[44]	Mar 2022	MI	IV 2a	Euclidean spatial alignment	CSP	transfer learning, logistic regression	Avg ACC: 77%	Preprocess, algorithm fusion
[45]	Mar 2022	ME	III 3a, III 4a, IV 2a	Filtering; multi basic methods; voting strategies, Special GAN		Deep ConvNet	ACC: III 3a 79%, III 4a 76%, IV 2a 66%	data augmentation, deep learning
[46]	Mar 2022	MI	own dataset, IV 1	segmentation, down-sampling, filtering	FFT + UMLDA (Uncorrelated Multilinear Discriminant Analysis) + CSP (Tensor-Based Frequency Features Combination, TFFC)	SVM	ACC: own 70.61%, IV 1 88.79%	Feature extraction fusion
[47]	Mar 2022	P300	Home Control	Downsample, filtering		EEG -TCFNet	Subject-dependent ACC: 91.2%; subject-independent ACC: 68.2%	Deep learning algorithm fusion
[48]	Apr 2022		DEAP	filtering	BGWO, Hurst index, WPD	Bi-LSTM	ACC: Valencia 99.45%; arousal 96.67%; liking 99.68%	Feature extraction fusion, Deep learning algorithms
[49]	Apr 2022	Visual stimuli	dataset from Stanford Digital Repository	AEP, Interpolation, boundary clipping		EEG-Conv-Transformer (improved Conv-Transformer)	ACC: 88.78% for 4 heads, 89.64% for 12 heads	Deep learning algorithm fusion
[50]	Apr 2022		STEW dataset	Filtering, Artifact Subspace Reconstruction, ICA		EEG-TNet (Time-Fixed Convolutional + BiLSTM)	ACC: 99.21% – 99.82%	Preprocessing, deep learning algorithm fusion

Table 1: Cont.

Ref.	Month and Year	Paradigm	Dataset	Pre-processing	Feature Extraction	Classification Algorithms	Indicators	Main Direction
[51]	Apr 2022	MI	II 3, III 3b, IV 2b	ACGAN, S transform, frequency slicing		CNN-ELM	For ACC: avg. 83.5%; II.3 up to 92.1% – 94.64%; III.3b avg 83.50%; IV.2b 78.2%	Data augmentation, Deep learning algorithm fusion
[52]	Apr 2022	P300	Spelling BCI competition III Dataset 2	time slicing, fitering		IncepA-EEGNet (Inception block + attention)	ACC 75.53 – 79.14, ITR 21.59 – 33.44 bits/min	Deep learning algorithm fusion
[53]	May 2022	MI	IV 2a, High Gamma Dataset	sliding window for DA		DSC-ConvLSTM	ACC: 73.7% in IV 2a, 92.6% in HGD	data augmentation; deep learning algorithm fusion
[54]	Jun 2022		SEED, DEAP	filtering, downsampling, SLORETA		Bayes + DGCNN	ACC: 99.25%	preprocessing, deep learning fusion
[55]	Jun 2022	P300	IV 2a	EA alignment, channel selection		attention + CNN	ACC: 86.03%	preprocessing, channel selection, deep learning fusion
[56]	Jun 2022		DEAP	filtering, ICA, sliding window		CNN	ACC: 95.15% for subject-dependence, 88.28% for subject-independence	deep learning fusion
[57]	Jun 2022	P300	own dataset	filtering, baseline correction		LR-CNN	ACC: 91.9% – 95.6%	deep learning fusion
[58]	Jun 2022	P300	own dataset	basic methods, ICA, segmentation		SAST-GCN	ACC: 90.55%	preprocessing, deep learning fusion
[59]	Jun 2022	c-VEP	c-VEP BCI Dataset	multi methods	Riemann	MDM	ACC: 90.39%	preprocessing + feature fusion
[60]	Jun 2022	P300	II 2b, III 2, own dataset	basic methods, channel selection (RSBSBL Algorithm)		BLDA	channel selection	
[61]	Jul 2022	MI	IV. 2a	LSTM-AE	CSP		ACC: 60.66% – 97.76%	innovative preprocess

References

[1] P. Gaur et al. “A Sliding Window Common Spatial Pattern for Enhancing Motor Imagery Classification in EEG-BCI”. In: *IEEE Transactions on Instrumentation and Measurement* 70 (2021), pp. 1–9.

[2] F. Qi et al. “Single-Trial EEG Classification via Orthogonal Wavelet Decomposition-Based Feature Extraction”. In: *Front Neurosci* 15 (2021). Qi, Feifei Wang, Wenlong Xie, Xiaofeng Gu, Zhenghui Yu, Zhu Liang Wang, Fei Li, Yuanqing Wu, Wei eng Switzerland 2021/11/02 Front Neurosci. 2021 Oct 13;15:715855. doi: 10.3389/fnins.2021.715855. eCollection 2021., p. 715855. ISSN: 1662-4548 (Print) 1662-453X (Linking). DOI: 10.3389/fnins.2021.715855. URL: https://www.ncbi.nlm.nih.gov/pubmed/34720854.

[3] H. Varsehi and S. M. P. Firoozabadi. “An EEG channel selection method for motor imagery based brain-computer interface and neurofeedback using Granger causality”. In: *Neural Netw* 133 (2021). Varsehi, Hesam Firoozabadi, S Mohammad P eng 2020/11/22 Neural Netw. 2021 Jan;133:193-206. doi: 10.1016/j.neunet.2020.11.0[illegible] Epub 2020 Nov 10., pp. 193–206. ISSN: 1879-2782 (Electronic) 0893-6080 (Linking). DOI: 10.1016/j.neunet.2020.11.002. URL: https://www.ncbi.nlm.nih.gov/pubmed/33220643.

[4] F. Xu et al. “A transfer learning framework based on motor imagery rehabilitation for stroke”. In: *Sci Rep* 11.1 (2021). Xu, Fangzhou Miao, Yunjing Sun, Yanan Guo, Dongju Xu, Jiali Wang, Yuandong Li, Jincheng Li, Han Dong, Gege Rong, Fenqi Leng, Jiancai Zhang, Yang eng Research Support, Non-U.S. Gov’t England 2021/10/07 Sci Rep. 2021 Oct 5;11(1):19783. doi: 10.1038/s41598-021-99114-1., p. 19783. ISSN: 2045-2322 (Electronic) 2045-2322 (Linking). DOI: 10.1038/s41598-021-99114-1. URL: https://www.ncbi.nlm.nih.gov/pubmed/34611209.

[5] Y. Zhang et al. “Emergency Braking Intention Detect System Based on K-Order Propagation Number Algorithm: A Network Perspective”. In: *Brain Sci* 11.11 (2021). Zhang, Yuhong Liao, Yuan Zhang, Yudi Huang, Liya eng 61977039/National Natural Science Foundation of China Switzerland 2021/11/28 Brain Sci. 2021 Oct 27;11(11). pii: brainsci11111424. doi: 10.3390/brainsci11111424. ISSN: 2076-3425 (Print) 2076-3425 (Linking). DOI: 10.3390/brainsci11111424. URL: https://www.ncbi.nlm.nih.gov/pubmed/34827420.

[6] S. Lian et al. “A Novel Time-Incremental End-to-End Shared Neural Network with Attention-Based Feature Fusion for Multiclass Motor Imagery Recognition”. In: *Comput Intell Neurosci* 2021 (2021). Lian, Shidong Xu, Jialin Zuo, Guokun Wei, Xia Zhou, Huilin eng 2021/03/09 Comput Intell Neurosci. 2021 Feb 17;2021:6613105. doi: 10.1155/2021/6613105. eCollection 2021., p. 6613105. ISSN: 1687-5273 (Electronic). DOI: 10.1155/2021/6613105. URL: https://www.ncbi.nlm.nih.gov/pubmed/33679965.

[7] Eda Dagdevir and Mahmut Tokmakci. “Optimization of preprocessing stage in EEG based BCI systems in terms of accuracy and timing cost”. In: *Biomedical Signal Processing and Control* 67 (2021). ISSN: 17468094. DOI: 10.1016/j.bspc.2021.102548.

[8] M. Rashid et al. “The classification of motor imagery response: an accuracy enhancement through the ensemble of random subspace k-NN”. In: *PeerJ Comput Sci* 7 (2021). Rashid, Mamunur Bari, Bifta Sama Hasan, Md Jahid Razman, Mohd Azraai Mohd Musa, Rabiu Muazu Ab Nasir, Ahmad Fakhri P P Abdul Majeed, Anwar eng 2021/04/06 PeerJ Comput Sci. 2021 Mar 2;7:e374. doi: 10.7717/peerj-cs.374. eCollection 2021., e374. ISSN: 2376-5992 (Electronic) 2376-5992 (Linking). DOI: 10.7717/peerj-cs.374. URL: https://www.ncbi.nlm.nih.gov/pubmed/33817022.

[9] Y. Du, Z. Liu, and Z. Fu. “Motion Imagery Classification Algorithm Research Based on Hybrid Transfer Learning and Application in Brain-computer Interface”. In: *ACTA METROLOGICA SINICA* 45(5) (2021), pp. 629–637.

[10] T. Liu and D. Yang. “A three-branch 3D convolutional neural network for EEG-based different hand movement stages classification”. In: *Sci Rep* 11.1 (2021). Liu, Tianjun Yang, Deling eng Research Support, Non-U.S. Gov’t England 2021/05/26 Sci Rep. 2021 May 24;11(1):10758. doi: 10.1038/s41598-021-89414-x., p. 10758. ISSN: 2045-2322 (Electronic) 2045-2322 (Linking). DOI: 10.1038/s41598-021-89414-x. URL: https://www.ncbi.nlm.nih.gov/pubmed/34031436.

[11] X. Yin et al. “Optimal channel-based sparse time-frequency blocks common spatial pattern feature extraction method for motor imagery classification”. In: *Math Biosci Eng* 18.4 (2021). Yin, Xu Meng, Ming She, Qingshan Gao, Yunyuan Luo, Zhizeng eng Research Support, Non-U.S. Gov’t 2021/07/03 Math Biosci Eng. 2021 May 17;18(4):4247-4263. doi: 10.3934/mbe.2021213., pp. 4247–4263. ISSN: 1551-0018 (Electronic) 1547-1063 (Linking). DOI: 10.3934/mbe.2021213. URL: https://www.ncbi.nlm.nih.gov/pubmed/34198435.

[12] K. Zhang et al. “Weak Feature Extraction and Strong Noise Suppression for SSVEP-EEG Based on Chaotic Detection Technology”. In: *IEEE Trans Neural Syst Rehabil Eng* 29 (2021). Zhang, Kai Xu, Guanghua Du, Chenghang Wu, Yongchen Zheng, Xiaowei Zhang, Sicong Han, Chengcheng Liang, Renhao Chen, Ruiquan eng Research Support, Non-U.S. Gov’t 2021/04/20 IEEE Trans Neural Syst Rehabil Eng. 2021;29:862-871. doi: 10.1109/TNSRE.2021.3073918. Epub 2021 May 13., pp. 862–871. ISSN: 1558-0210 (Electronic) 1534-4320 (Linking). DOI: 10.1109/TNSRE.2021.3073918. URL: https://www.ncbi.nlm.nih.gov/pubmed/33872154.

[13] Z. Cai et al. “Cross-subject electroencephalogram emotion recognition based on maximum classifier discrepancy”. In: *Journal of Biomedical Engineering* 38.3 (2021). Cai, Ziliang Guo, Miaomiao Yang, Xinsheng Chen, Xintong Xu, Guizhi chi China 2021/06/29 Sheng Wu Yi Xue Gong Cheng Xue Za Zhi. 2021 Jun 25;38(3):455-462. doi: 10.7507/1001-5515.202012027., pp. 455–462. ISSN: 1001-5515 (Print) 1001-5515 (Linking). DOI: 10.7507/1001-5515.202012027. URL: https://www.ncbi.nlm.nih.gov/pubmed/34180190.

[14] D. De Venuto and G. Mezzina. “A Single-Trial P300 Detector Based on Symbolized EEG and Autoencoded-(1D)CNN to Improve ITR Performance in BCIs”. In: *Sensors (Basel)* 21.12 (2021). De Venuto, Daniela Mezzina, Giovanni eng Switzerland 2021/07/03 Sensors (Basel). 2021 Jun 8;21(12). pii: s21123961. doi: 10.3390/s21123961. ISSN: 1424-8220 (Electronic) 1424-8220 (Linking). DOI: 10.3390/s21123961. URL: https://www.ncbi.nlm.nih.gov/pubmed/34201381.

[15] C. Liu and J. Jin. “JI YU SHUANG PU DE WEN TAI SHI JUE YOU FA DIAN WEI NAO JI JIE KOU DAO LIAN XUAN ZE SUAN FA”. In: *REN GONG ZHI NENG(Artificial Intelligence)* 6 (2021), pp. 52–60. DOI: 10.16453/j.cnki.ISSN2096-5036.2021.06.006.

[16] J. Sun et al. “Classification algorithms of error-related potentials in brain-computer interface”. In: *Sheng Wu Yi Xue Gong Cheng Xue Za Zhi* 38.3 (2021). Sun, Jinsong Jung, Tzyy-Ping Xiao, Xiaolin Meng, Jiayuan Xu, Minpeng Ming, Dong chi China 2021/06/29 Sheng Wu Yi Xue Gong Cheng Xue Za Zhi. 2021 Jun 25;38(3):463-472. doi: 10.7507/1001-5515.202012013., pp. 463–472. ISSN: 1001-5515 (Print) 1001-5515 (Linking). DOI: 10.7507/1001-5515.202012013. URL: https://www.ncbi.nlm.nih.gov/pubmed/34180191.

[17] E. Lashgari et al. “An end-to-end CNN with attentional mechanism applied to raw EEG in a BCI classification task”. In: *J Neural Eng* 18.4 (2021). Lashgari, Elnaz Ott, Jordan Connelly, Akima Baldi, Pierre Maoz, Uri eng Research Support, Non-U.S. Gov’t England 2021/08/06 J Neural Eng. 2021 Aug 25;18(4). doi: 10.1088/1741-2552/ac1ade. ISSN: 1741-2552 (Electronic) 1741-2552 (Linking). DOI: 10.1088/1741-2552/ac1ade. URL: https://www.ncbi.nlm.nih.gov/pubmed/34352734.

[18] J. Liu, F. Ye, and H. Xiong. “Multi-class motor imagery EEG classification method with high accuracy and low individual differences based on hybrid neural network”. In: *J Neural Eng* 18.4 (2021). Liu, Jinzhen Ye, Fangfang Xiong, Hui eng Research Support, Non-U.S. Gov’t England 2021/08/19 J Neural Eng. 2021 Aug 31;18(4). doi: 10.1088/1741-2552/ac1ed0. ISSN: 1741-2552 (Electronic) 1741-2552 (Linking). DOI: 10.1088/1741-2552/ac1ed0. URL: https://www.ncbi.nlm.nih.gov/pubmed/34407527.

[19] F. M. Aellen et al. “Convolutional neural networks for decoding electroencephalography responses and visualizing trial by trial changes in discriminant features”. In: *J Neurosci Methods* 364 (2021). Aellen, Florence M Goktepe-Kavis, Pinar Apostolopoulos, Stefanos Tzovara, Athina eng Research Support, Non-U.S. Gov’t Netherlands 2021/09/27 J Neurosci Methods. 2021 Dec 1;364:109367. doi: 10.1016/j.jneumeth.2021.109367. Epub 2021 Sep 23., p. 109367. ISSN: 1872-678X (Electronic) 0165-0270 (Linking). DOI: 10.1016/j.jneumeth.2021.109367. URL: https://www.ncbi.nlm.nih.gov/pubmed/34563599%20https://www.sciencedirect.com/science/article/pii/S0165027021003022?via%5C%3Dihub.

[20] Ashwini S. R and H. C. Nagaraj. “Classification of EEG signal using EACA based approach at SSVEP-BCI”. In: *IAES International Journal of Artificial Intelligence (IJ-AI)* 10.3 (2021). ISSN: 2252-8938 2089-4872. DOI: 10.11591/ijai.v10.i3.pp717-726.

[21] M. A. Awais et al. “Effective Connectivity for Decoding Electroencephalographic Motor Imagery Using a Probabilistic Neural Network”. In: *Sensors (Basel)* 21.19 (2021). Awais, Muhammad Ahsan Yusoff, Mohd Zuki Khan, Danish M Yahya, Norashikin Kamel, Nidal Ebrahim, Mansoor eng er 015ME0-2/Iqra University Switzerland 2021/10/14 Sensors (Basel). 2021 Sep 30;21(19). pii: s21196570. doi: 10.3390/s21196570. ISSN: 1424-8220 (Electronic) 1424-8220 (Linking). DOI: 10.3390/s21196570. URL: https://www.ncbi.nlm.nih.gov/pubmed/34640888.

[22] N. Gao, Z. Gao, and H. Zhang. “Riemannian approach research for the feature extraction and classification of motor imagery electroencephalogram(EEG) signals”. In: *Journal of Biomedical Engineering Research* 40(3) (2021), pp. 246–251.

[23] Q. Wang and H. Quan. "Research on the classification of motor imagery EEG by optimized SVM based surface-simplex swarm evolution". In: *Journal of Electronic Measurement and Instrumentation* 35(9) (2021), pp. 157–163.

[24] Babak Asheri et al. "Enhancing detection of steady-state visual evoked potentials using frequency and harmonics of that frequency in OpenVibe". In: *Biomedical Engineering Advances* 2 (2021). ISSN: 26670992. DOI: 10.1016/j.bea.2021.100022.

[25] Zhongke Gao et al. "Classification of EEG Signals on VEP-Based BCI Systems With Broad Learning". In: *IEEE Transactions on Systems, Man, and Cybernetics: Systems* 51.11 (2021), pp. 7143–7151. ISSN: 2168-2216 2168-2232. DOI: 10.1109/tsmc.2020.2964684.

[26] Xiaozhong Geng et al. "An improved feature extraction algorithms of EEG signals based on motor imagery brain-computer interface". In: *Alexandria Engineering Journal* 61.6 (2022), pp. 4807–4820. ISSN: 11100168. DOI: 10.1016/j.aej.2021.10.034.

[27] D. Vorontsova et al. "Silent EEG-Speech Recognition Using Convolutional and Recurrent Neural Network with 85% Accuracy of 9 Words Classification". In: *Sensors (Basel)* 21.20 (2021). Vorontsova, Darya Menshikov, Ivan Zubov, Aleksandr Orlov, Kirill Rikunov, Peter Zvereva, Ekaterina Flitman, Lev Lanikin, Anton Sokolova, Anna Markov, Sergey Bernadotte, Alexandra eng Switzerland 2021/10/27 Sensors (Basel). 2021 Oct 11;21(20). pii: s21206744. doi: 10.3390/s21206744. ISSN: 1424-8220 (Electronic) 1424-8220 (Linking). DOI: 10.3390/s21206744. URL: https://www.ncbi.nlm.nih.gov/pubmed/34695956.

[28] Md Ferdous Wahid and Reza Tafreshi. "Improved Motor Imagery Classification Using Regularized Common Spatial Pattern with Majority Voting Strategy". In: *IFAC-PapersOnLine* 54.20 (2021), pp. 226–231. ISSN: 2405-8963. DOI: https://doi.org/10.1016/j.ifacol.2021.11.179..

[29] W. Fan et al. "Support Vector Machine Algorithm with Multi-Sample Fusion for P300 Signal Classification". In: *Journal of Wuhan Institute of Technology* 43(6) (2021), pp. 670–674. DOI: 10.19843/j.cnki.CN42-1779/TQ.202101006.

[30] F. Ferracuti et al. "Comparing between Different Sets of Preprocessing, Classifiers, and Channels Selection Techniques to Optimise Motor Imagery Pattern Classification System from EEG Pattern Recognition". In: *Brain Sci* 12.1 (2021). Ferracuti, Francesco Iarlori, Sabrina Mansour, Zahra Monteriu, Andrea Porcaro, Camillo eng Switzerland 2022/01/22 Brain Sci. 2021 Dec 31;12(1). pii: brainsci12010057. doi: 10.3390/brainsci12010057. ISSN: 2076-3425 (Print) 2076-3425 (Linking). DOI: 10.3390/brainsci12010057. URL: https://www.ncbi.nlm.nih.gov/pubmed/35053801.

[31] Md Nahidul Islam et al. "A hybrid scheme for AEP based hearing deficiency diagnosis: CWT and convoluted k-nearest neighbour (CKNN) pipeline". In: *Neuroscience Informatics* 2.1 (2022). ISSN: 27725286. DOI: 10.1016/j.neuri.2021.100037.

[32] C. Xu, H. Zhang, and L. Sun. "Prediction of hand grip motion intention based on sample entropy and time-frequency analysis". In: *Journal of Zhejiang University (Engineering Science)* 55(12) (2021), pp. 2315–2322. DOI: 10.3785/j.issn.1008-973X.2021.12.011.

[33] Tingting Jia, Chaoyi Dong, and Shuang Ma. "Brain-computer interface of motor imaging based on mutual information feature extraction". In: *Chinese Journal of Medical Physics* 39(1) (2022), pp. 63–68. DOI: 10.3969/j.issn.1005-202X.2022.01.011.

[34] S. Kim et al. "Enhanced Recognition of Amputated Wrist and Hand Movements by Deep Learning Method Using Multimodal Fusion of Electromyography and Electroencephalography". In: *Sensors (Basel)* 22.2 (2022). Kim, Sehyeon Shin, Dae Youp Kim, Taekyung Lee, Sangsook Hyun, Jung Keun Park, Sung-Min eng 2020R1A6A1A03047902/National Research Foundation of Korea 2020R1A2C2005385, 2020R1A2C2004764/Korea government (Ministry of Science and ICT, MSIT) 202017D01/Korean government (MSIT, the Ministry of Trade, Industry and Energy, the Ministry of Health & Welfare, and the Ministry of Food and Drug Safety) Switzerland 2022/01/23 Sensors (Basel). 2022 Jan 16;22(2). pii: s22020680. doi: 10.3390/s22020680. ISSN: 1424-8220 (Electronic) 1424-8220 (Linking). DOI: 10.3390/s22020680. URL: https://www.ncbi.nlm.nih.gov/pubmed/35062641.

[35] H. Li, M. Ding, and R. Zhang. "Motor Imaginative EEG Classification Algorithm Based on Feature Fusion Neural Network". In: *Chinese Journal of Medical Physics* 39(1) (2022), pp. 69–75. DOI: 10.3969/j.issn.1005-202X.2022.01.012.

[36] F. Mattioli, C. Porcaro, and G. Baldassarre. "A 1D CNN for high accuracy classification and transfer learning in motor imagery EEG-based brain-computer interface". In: *J Neural Eng* 18.6 (2022). Mattioli, F Porcaro, C Baldassarre, G eng Research Support, Non-U.S. Gov't England 2021/12/18 J Neural Eng. 2022 Jan 6;18(6). doi: 10.1088/1741-2552/ac4430. ISSN: 1741-2552 (Electronic) 1741-2552 (Linking). DOI: 10.1088/1741-2552/ac4430. URL: https://www.ncbi.nlm.nih.gov/pubmed/34920443.

[37] Nazmi Sofian Suhaimi, James Mountstephens, and Jason Teo. “A Dataset for Emotion Recognition Using Virtual Reality and EEG (DER-VREEG): Emotional State Classification Using Low-Cost Wearable VR-EEG Headsets”. In: *Big Data and Cognitive Computing* 6.1 (2022). ISSN: 2504-2289. DOI: 10.3390/bdcc6010016.

[38] Yuan Tang et al. “Motor Imagery EEG Decoding Based on New Spatial-Frequency Feature and Hybrid Feature Selection Method”. In: *Mathematical Problems in Engineering* 2022 (2022), pp. 1–12. ISSN: 1563-5147 1024-123X. DOI: 10.1155/2022/2856818.

[39] Y. Cui et al. “A spatial-temporal hybrid feature extraction method for rapid serial visual presentation of electroencephalogram signals”. In: *Chinese Journal of Biomedical Engineering* 39.1 (2022). Cui, Yujie Xie, Songyun Xie, Xinzhou Duan, Xu Gao, Chuanlin chi China 2022/03/02 Sheng Wu Yi Xue Gong Cheng Xue Za Zhi. 2022 Feb 25;39(1):39-46. doi: 10.7507/1001-5515.202104049., pp. 39–46. ISSN: 1001-5515 (Print) 1001-5515 (Linking). DOI: 10.7507/1001-5515.202104049. URL: https://www.ncbi.nlm.nih.gov/pubmed/35231964.

[40] Yanfei Lin, Boyu Zang, and Rongxiao Guo. “A Deep Learning Method for SSVEP Classification Based on Phase and Frequency Characteristics”. In: *Journal of Electronics& Information Technology* 44(2) (2022), pp. 446–454. DOI: 10.11999/JEIT210816.

[41] P. Ma et al. “A classification algorithm of an SSVEP brain-Computer interface based on CCA fusion wavelet coefficients”. In: *J Neurosci Methods* 371 (2022). Ma, Pengfei Dong, Chaoyi Lin, Ruijing Ma, Shuang Jia, Tingting Chen, Xiaoyan Xiao, Zhiyun Qi, Yongsheng eng Research Support, Non-U.S. Gov’t Netherlands 2022/02/14 J Neurosci Methods. 2022 Apr 1;371:109502. doi: 10.1016/j.jneumeth.2022.109502. Epub 2022 Feb 11., p. 109502. ISSN: 1872-678X (Electronic) 0165-0270 (Linking). DOI: 10.1016/j.jneumeth.2022.109502. URL: https://www.ncbi.nlm.nih.gov/pubmed/35151665.

[42] M. Meng, Z. Dong, and Y. Gao. “Correlation and Sparse Representation Based Channel Selection of Motor Imagery Electroencephalogram”. In: *Journal of Electronics & Information Technology* 44(2) (2022), pp. 477–485. DOI: 10.11999/JEIT210778.

[43] O. Ali et al. “Enhancing the decoding accuracy of EEG signals by the introduction of anchored-STFT and adversarial data augmentation method”. In: *Sci Rep* 12.1 (2022). Ali, Omair Saif-Ur-Rehman, Muhammad Dyck, Susanne Glasmachers, Tobias Iossifidis, Ioannis Klaes, Christian eng Research Support, Non-U.S. Gov’t England 2022/03/12 Sci Rep. 2022 Mar 10;12(1):4245. doi: 10.1038/s41598-022-07992-w., p. 4245. ISSN: 2045-2322 (Electronic) 2045-2322 (Linking). DOI: 10.1038/s41598-022-07992-w. URL: https://www.ncbi.nlm.nih.gov/pubmed/35273310.

[44] Li Chen, Anmin Gong, and Peng Ding. “EEG signal decoding of motor imagination based on euclidean space-weighted logistic regression transfer learning”. In: *JOURNAL OF NANJING UNIVERSITY(NATURAL SCIENCE)* 58(2) (2022), pp. 264–274. DOI: 1010.13232/j.

[45] W. Ko et al. “Semi-supervised generative and discriminative adversarial learning for motor imagery-based brain-computer interface”. In: *Sci Rep* 12.1 (2022). Ko, Wonjun Jeon, Eunjin Yoon, Jee Seok Suk, Heung-Il eng Research Support, Non-U.S. Gov’t England 2022/03/19 Sci Rep. 2022 Mar 17;12(1):4587. doi: 10.1038/s41598-022-08490-9., p. 4587. ISSN: 2045-2322 (Electronic) 2045-2322 (Linking). DOI: 10.1038/s41598-022-08490-9. URL: https://www.ncbi.nlm.nih.gov/pubmed/35301366.

[46] Y. Pei et al. “A Tensor-Based Frequency Features Combination Method for Brain-Computer Interfaces”. In: *IEEE Trans Neural Syst Rehabil Eng* 30 (2022). Pei, Yu Luo, Zhiguo Zhao, Hongyu Xu, Dengke Li, Weiguo Yan, Ye Yan, Huijiong Xie, Liang Xu, Minpeng Yin, Erwei eng Research Support, Non-U.S. Gov’t 2021/11/05 IEEE Trans Neural Syst Rehabil Eng. 2022;30:465-475. doi: 10.1109/TNSRE.2021.3125386. Epub 2022 Mar 8., pp. 465–475. ISSN: 1558-0210 (Electronic) 1534-4320 (Linking). DOI: 10.1109/TNSRE.2021.3125386. URL: https://www.ncbi.nlm.nih.gov/pubmed/34735347.

[47] Christian Flores Vega et al. “Fuzzy temporal convolutional neural networks in P300-based Brain-computer interface for smart home interaction”. In: *Applied Soft Computing* 117 (2022), p. 108359.

[48] M. Algarni et al. “Deep Learning-Based Approach for Emotion Recognition Using Electroencephalography (EEG) Signals Using Bi-Directional Long Short-Term Memory (Bi-LSTM)”. In: *Sensors (Basel)* 22.8 (2022). Algarni, Mona Saeed, Faisal Al-Hadhrami, Tawfik Ghabban, Fahad Al-Sarem, Mohammed eng 77 /442/The Deputyship for Research & Innovation, Ministry of Education in Saudi Arabia Switzerland 2022/04/24 Sensors (Basel). 2022 Apr 13;22(8). pii: s22082976. doi: 10.3390/s22082976. ISSN: 1424-8220 (Electronic) 1424-8220 (Linking). DOI: 10.3390/s22082976. URL: https://www.ncbi.nlm.nih.gov/pubmed/35458962.

[49] Subhranil Bagchi and Deepti R.Bathula. “EEG-ConvTransformer for Single-Trial EEG based Visual Stimulus Classification”. In: *Pattern Recognition* 129.0031-3203 (2022), p. 108757. DOI: https://doi.org/10.1016/j.patcog.2022.108757.

[50] C. Fan et al. “EEG-TNet: An End-To-End Brain Computer Interface Framework for Mental Workload Estimation”. In: *Front Neurosci* 16 (2022). Fan, Chaojie Hu, Jin Huang, Shufang Peng, Yong Kwong, Sam eng Switzerland 2022/05/17 Front Neurosci. 2022 Apr 25;16:869522. doi: 10.3389/fnins.2022.869522. eCollection 2022., p. 869522. ISSN: 1662-4548 (Print) 1662-453X (Linking). DOI: 10.3389/fnins.2022.869522. URL: https://www.ncbi.nlm.nih.gov/pubmed/35573313.

[51] C. Song, Y. Sheng, and Z. Ning. “Deep learning-based method for recognition of motion imagery EEG signal”. In: *Transducer and Microsystem Technologies* 41(4) (2022), pp. 125–133.

[52] M. Xu et al. “IncepA-EEGNet: P300 signal detection method based on fusion of Inception network and attention mechanism”. In: *Journal of Zhejiang University (Engineering Science)* 56(4) (2022), pp. 745–753. DOI: 10.3785/j.issn.1008-973X.2022.04.014.

[53] L. Li and N. Sun. “Attention-Based DSC-ConvLSTM for Multiclass Motor Imagery Classification”. In: *Comput Intell Neurosci* 2022 (2022). Li, Li Sun, Nan eng 2022/05/17 Comput Intell Neurosci. 2022 May 5;2022:8187009. doi: 10.1155/2022/8187009. eCollection 2022., p. 8187009. ISSN: 1687-5273 (Electronic). DOI: 10.1155/2022/8187009. URL: https://www.ncbi.nlm.nih.gov/pubmed/35571721.

[54] S. Asadzadeh et al. “Accurate emotion recognition using Bayesian model based EEG sources as dynamic graph convolutional neural network nodes”. In: *Sci Rep* 12.1 (2022). Asadzadeh, Shiva Yousefi Rezaii, Tohid Beheshti, Soosan Meshgini, Saeed eng England 2022/06/19 Sci Rep. 2022 Jun 18;12(1):10282. doi: 10.1038/s41598-022-14217-7., p. 10282. ISSN: 2045-2322 (Electronic) 2045-2322 (Linking). DOI: 10.1038/s41598-022-14217-7. URL: https://www.ncbi.nlm.nih.gov/pubmed/35717542.

[55] Zhanyuan Chang, Congcong Zhang, and Chuanjiang Li. “Motor Imagery EEG Classification Based on Transfer Learning and Multi-Scale Convolution Network”. In: *Micromachines* 13.6 (2022). ISSN: 2072-666X. DOI: 10.3390/mi13060927.

[56] J. Chen et al. “Electroencephalograph-Based Emotion Recognition Using Brain Connectivity Feature and Domain Adaptive Residual Convolution Model”. In: *Front Neurosci* 16 (2022). Chen, Jingxia Min, Chongdan Wang, Changhao Tang, Zhezhe Liu, Yang Hu, Xiuwen eng Switzerland 2022/07/12 Front Neurosci. 2022 Jun 22;16:878146. doi: 10.3389/fnins.2022.878146. eCollection 2022., p. 878146. ISSN: 1662-4548 (Print) 1662-453X (Electronic) 1662-453X (Linking). DOI: 10.3389/fnins.2022.878146. URL: https://www.ncbi.nlm.nih.gov/pubmed/35812226.

[57] Q. Li et al. “A P300-Detection Method Based on Logistic Regression and a Convolutional Neural Network”. In: *Front Comput Neurosci* 16 (2022). Li, Qi Wu, Yan Song, Yu Zhao, Di Sun, Meiqi Zhang, Zhilin Wu, Jinglong eng Switzerland 2022/07/06 Front Comput Neurosci. 2022 Jun 16;16:909553. doi: 10.3389/fncom.2022.909553. eCollection 2022., p. 909553. ISSN: 1662-5188 (Print) 1662-5188 (Electronic) 1662-5188 (Linking). DOI: 10.3389/fncom.2022.909553. URL: https://www.ncbi.nlm.nih.gov/pubmed/35782086.

[58] R. Lu et al. “SAST-GCN: Segmentation Adaptive Spatial Temporal-Graph Convolutional Network for P3-Based Video Target Detection”. In: *Front Neurosci* 16 (2022). Lu, Runnan Zeng, Ying Zhang, Rongkai Yan, Bin Tong, Li eng Switzerland 2022/06/21 Front Neurosci. 2022 Jun 2;16:913027. doi: 10.3389/fnins.2022.913027. eCollection 2022., p. 913027. ISSN: 1662-4548 (Print) 1662-453X (Electronic) 1662-453X (Linking). DOI: 10.3389/fnins.2022.913027. URL: https://www.ncbi.nlm.nih.gov/pubmed/35720707.

[59] J. Ying, Q. Wei, and X. Zhou. “Riemannian geometry-based transfer learning for reducing training time in c-VEP BCIs”. In: *Sci Rep* 12.1 (2022). Ying, Jiahui Wei, Qingguo Zhou, Xichen eng Research Support, Non-U.S. Gov't England 2022/06/15 Sci Rep. 2022 Jun 14;12(1):9818. doi: 10.1038/s41598-022-14026-y., p. 9818. ISSN: 2045-2322 (Electronic) 2045-2322 (Linking). DOI: 10.1038/s41598-022-14026-y. URL: https://www.ncbi.nlm.nih.gov/pubmed/35701505.

[60] X. Zhao et al. “A Regional Smoothing Block Sparse Bayesian Learning Method With Temporal Correlation for Channel Selection in P300 Speller”. In: *Front Hum Neurosci* 16 (2022). Zhao, Xueqing Jin, Jing Xu, Ren Li, Shurui Sun, Hao Wang, Xingyu Cichocki, Andrzej eng Switzerland 2022/06/28 Front Hum Neurosci. 2022 Jun 10;16:875851. doi: 10.3389/fnhum.2022.875851. eCollection 2022., p. 875851. ISSN: 1662-5161 (Print) 1662-5161 (Electronic) 1662-5161 (Linking). DOI: 10.3389/fnhum.2022.875851. URL: https://www.ncbi.nlm.nih.gov/pubmed/35754766.

[61] N. Ayoobi and E. B. Sadeghian. “Unsupervised Motor Imagery Saliency Detection Based on Self-Attention Mechanism”. In: *2022 44th Annual International Conference of the IEEE Engineering in Medicine & Biology Society (EMBC)*, pp. 4817–4820. ISBN: 2694-0604. DOI: 10.1109/EMBC48229.2022.9871906.
